# Practical Imaging Interpretation in Patients Suspected of Having Idiopathic Pulmonary Fibrosis: Official Recommendations from the Radiology Working Group of the Pulmonary Fibrosis Foundation

**DOI:** 10.1148/ryct.2021200279

**Published:** 2021-02-25

**Authors:** Stephen Hobbs, Jonathan H. Chung, Jay Leb, Kate Kaproth-Joslin, David A. Lynch

**Affiliations:** From the Department of Radiology, University of Kentucky, 800 Rose St, HX-315B, Lexington, KY 40536 (S.H.); Department of Radiology, University of Chicago, Chicago, Ill (J.H.C.); Department of Radiology, Columbia University, New York, NY (J.L.); Department of Imaging Sciences, University of Rochester, Rochester, NY (K.K.J.); and Department of Radiology, National Jewish Health, Denver, Colo (D.A.L.).

## Abstract

Imaging serves a key role in the diagnosis of patients suspected of having idiopathic pulmonary fibrosis (IPF). Accurate pattern classification at thin-section chest CT is a key step in multidisciplinary discussions, guiding the need for surgical lung biopsy and determining available pharmacologic therapies. The recent approval of new treatments for fibrosing lung disease has made it more critical than ever for radiologists to facilitate accurate and early diagnosis of IPF. This document was developed by the Radiology Working Group of the Pulmonary Fibrosis Foundation with the goal of providing a practical guide for radiologists. In this review, the critical imaging patterns of IPF, pitfalls in imaging classifications, confounding imaging findings with other fibrotic lung diseases, and reporting standards for cases of lung fibrosis will be discussed.

Published under a CC BY 4.0 license.

See also the commentary by White and Galvin in this issue.

**Summary**

Understanding the imaging findings and patterns associated with idiopathic pulmonary fibrosis and other fibrotic lung diseases is a key task of radiologists that can substantially impact patient care.

**Essentials**

■ Thin-section CT including inspiratory, expiratory, and prone sequences is the most important imaging tool for the evaluation of pulmonary fibrosis.
■ Categorizing the imaging pattern of fibrosis according to the Fleischner Society guidelines and/or American Thoracic Society guidelines is a critical role for the radiologist.
■ Radiology reports should use recognized standard terminology and include supporting language to justify the fibrosis pattern diagnosed.
■ Multidisciplinary discussion is the reference standard for diagnosis of diffuse fibrotic lung disease, and imaging plays a key role in these discussions.

## Introduction

Interstitial lung disease (ILD) consists of several hundred separate diseases, each with sometimes unique, but frequently overlapping, patterns of lung injury at thin-section CT. This guide focuses on the key imaging findings and differentiators necessary to evaluate a patient for idiopathic pulmonary fibrosis (IPF). IPF is a progressive chronic interstitial fibrotic lung disease of unknown etiology (1). Risk factors include older age, family history, chronic gastrointestinal reflux, smoking, and some environmental exposures (2–6). Typical clinical manifestations are nonspecific and include chronic dyspnea, dry cough, fatigue, lower lung crackles at physical examination, and digital clubbing. Pulmonary function tests typically demonstrate restriction and reduced diffusion capacity (3,6). The average survival after diagnosis is 3–4 years with some patients progressing more rapidly or, more rarely, slowly, with extended survival times of more than a decade (3,6,7). Early diagnosis of IPF by using imaging is critically important due to the poor prognosis of the disease and to guide patient selection for usage of antifibrotic medications (pirfenidone and nintedanib) that can reduce decline in pulmonary function (3,6,8,9).

The Pulmonary Fibrosis Foundation has developed a network of care centers to promote excellence in the management of IPF and other fibrotic lung conditions. As the field of pulmonary fibrosis continues to grow, this foundation has established working groups to provide views on topics important to the diagnosis and treatment of pulmonary fibrosis using published data and expert opinion. The Pulmonary Fibrosis Foundation formed a Radiology Working Group to provide a practical guide for radiologic diagnosis of fibrotic lung disease, based on recent evidence-based recommendations (3,6).

## Imaging Modalities Used for the Assessment of ILD

### Chest Radiography

Chest radiography is frequently an initial screening test for ILD due to its wide availability and low radiation exposure to the patient. However, ILD, if mild or early in the disease course, is difficult to identify on chest radiographs. Normal radiographs were described in older literature (1970s) in 10%–15% of patients with ILD, and this number is probably higher in clinical practice today given advances in cross-sectional imaging to detect very mild or early disease (10). Characterizing ILD to the extent necessary to guide diagnosis and further management is also usually not possible on radiographs, and patients with obesity or underinflation can cause false-positive radiograph interpretation due to the perception of increased interstitial markings in those patients (2). Chest radiographs are still frequently used to evaluate patients with pulmonary fibrosis for alternative diagnoses such as pneumonia or pneumothorax.

### Thin-Section CT

Thin-section CT, in which images are reconstructed with thin sections and high-spatial-frequency algorithms, is recommended to optimally image the lung interstitium and to characterize parenchymal abnormalities. Technical factors used for the thin-section CT scanning protocol are important and can help prevent diagnostic errors when properly set (2,3,6). While high-spatial-resolution reconstruction is important, excessive edge enhancement can result in image noise that interferes with interpretation. In general, a moderate edge-enhancing reconstruction algorithm is preferred, such as Siemens B45f, GE Bone, Philips D or YB, and Toshiba Lung Std (3). Section thickness directly affects the degree of partial volume averaging, and as such, a thickness of 1.5 mm or less should be used. Using thicker sections results in loss of definition of small vessels, airways, and septa (Fig 1). Additionally, any reticular abnormality present will appear more ground glass in quality. Until the past 5–10 years, noncontiguous thin-section CT imaging was frequently performed. However, this should in general no longer be performed for inspiratory scanning with the now widespread availability of multidetector CT. Multidetector CT with volumetric thin section acquisition allows for contiguous visualization of the whole lung and greatly improves the diagnostic yield of thin-section CT. Multiplanar reconstructions, specifically coronal imaging, can be helpful in distinguishing distal bronchiolectasis from honeycomb cyst formation (2,3). Maximum intensity projection imaging can be helpful for detecting nodules, including the micronodules seen in diffuse lung diseases, and minimum intensity projections can be useful on occasion for improved identification of emphysema and air trapping (2). Filtered back projection has been the industry standard for CT image reconstruction, used to improve image quality while decreasing radiation dose. Unfortunately, in the setting of thin section thickness and high resolution, both of which are required for thin-section CT imaging, this reconstruction method leads to a low signal-to-noise ratio, which substantially limits image interpretation. The iterative reconstruction technique is less sensitive to image noise and therefore can improve image quality by reducing image noise and streak artifact and improving spatial resolution, even in the setting of thin section thickness and low radiation dose technique (11).

**Figure 1a:**
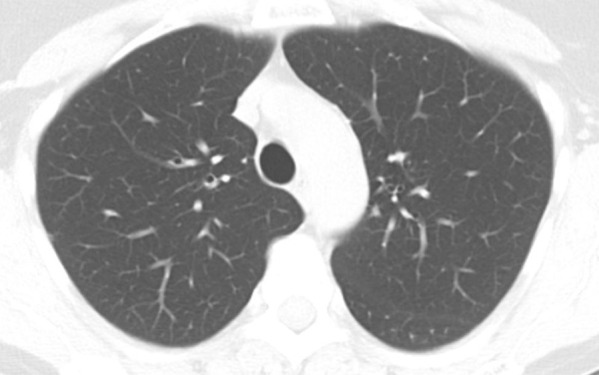
Axial thin-section CT scans in a healthy patient. **(a)** Inspiratory examination reconstructed with 5-mm thick sections and soft-tissue reconstruction demonstrates relative loss of definition of the interlobular septa and secondary pulmonary lobule. **(b)** Inspiratory sequence reconstructed with 1-mm thin sections and moderate edge-enhancing kernel reconstruction shows normal appearance. **(c)** Expiratory imaging shows bowing of the posterior tracheal wall and diffuse mildly heterogeneous increase in lung attenuation. Note that mild heterogeneous lung attenuation is normal.

**Figure 1b:**
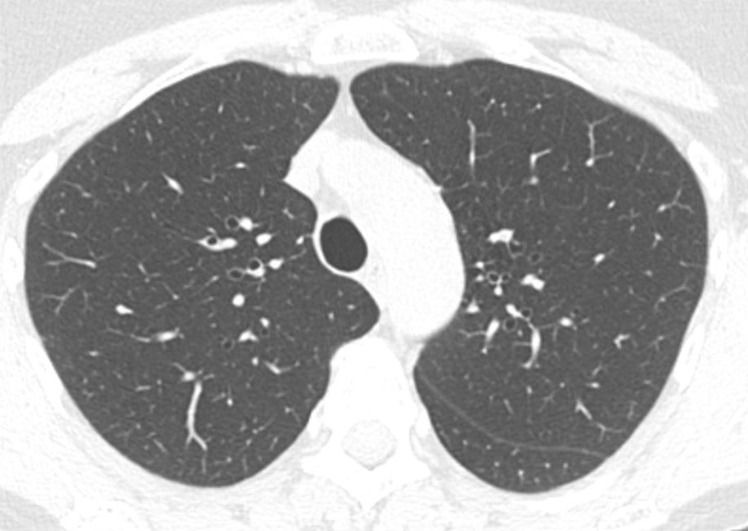
Axial thin-section CT scans in a healthy patient. **(a)** Inspiratory examination reconstructed with 5-mm thick sections and soft-tissue reconstruction demonstrates relative loss of definition of the interlobular septa and secondary pulmonary lobule. **(b)** Inspiratory sequence reconstructed with 1-mm thin sections and moderate edge-enhancing kernel reconstruction shows normal appearance. **(c)** Expiratory imaging shows bowing of the posterior tracheal wall and diffuse mildly heterogeneous increase in lung attenuation. Note that mild heterogeneous lung attenuation is normal.

**Figure 1c:**
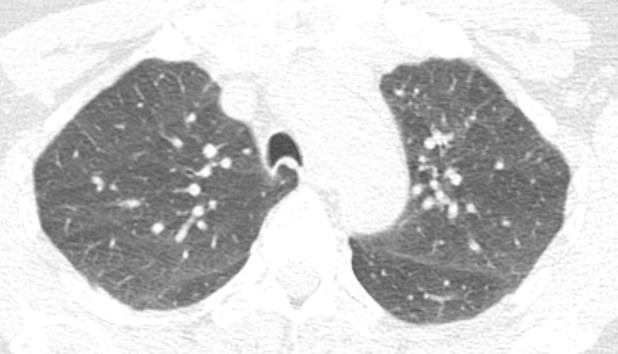
Axial thin-section CT scans in a healthy patient. **(a)** Inspiratory examination reconstructed with 5-mm thick sections and soft-tissue reconstruction demonstrates relative loss of definition of the interlobular septa and secondary pulmonary lobule. **(b)** Inspiratory sequence reconstructed with 1-mm thin sections and moderate edge-enhancing kernel reconstruction shows normal appearance. **(c)** Expiratory imaging shows bowing of the posterior tracheal wall and diffuse mildly heterogeneous increase in lung attenuation. Note that mild heterogeneous lung attenuation is normal.

Because the fine detail of the lung parenchyma is of paramount importance in thin-section CT scans, technologists should review scans immediately after acquisition and consider repeat scanning for any portions of the study that are compromised by patient or respiratory motion or inadequate inspiration (2,3). Respiratory motion (and sometimes unavoidable cardiac motion) can produce pulsation or star artifact, particularly in the lingula. These motions can result in thin streaks mimicking reticular abnormality or doubling artifacts mimicking bronchiectasis. Inadequate inspiration can simulate ground-glass abnormalities and make evaluation of the lung bases difficult (2,3). Proper patient coaching on inspiratory and expiratory effort, such as described by Bankier et al, can increase the likelihood of obtaining adequate imaging (12).

In addition to inspiratory acquisitions, two additional scans—expiratory and prone acquisitions—are considered part of a “complete” thin-section CT. Unlike the inspiratory examination, the expiratory and prone images may be acquired with noncontiguous parameters to minimize radiation dose (10–20 mm of space between axial sections) (2,3).

Expiratory sequences are specifically included to evaluate the presence of small airways disease. Air trapping at expiratory thin-section CT has been shown to correlate with obstructive deficits at pulmonary function testing, and the presence of small airways disease can substantially change the differential diagnosis (see discussion on mosaic attenuation). Ensuring that an adequate expiratory examination was performed is important to avoid false-negative findings. Proper CT technologist training and patient preparation can facilitate good expiratory scans. Expected CT findings on expiratory sequence include increased lung attenuation and decreased overall lung cross-sectional area. Additionally, anterior bowing of the posterior tracheal wall (the noncartilaginous portion) is frequently one of the easiest and most reliable signs of expiratory effort (Fig 1). If the tracheal lumen does not change between inspiration and expiration, the validity of the expiratory effort should be questioned (2,3). If the degree of anterior bowing of the tracheal wall is greater than 50%–70%, then this could reflect underlying tracheomalacia or excessive dynamic airways collapse.

Prone imaging is necessary in some patients to detect early or mild ILD. Dependent lung atelectasis is often present at supine inspiratory scanning, which can mimic mild subpleural reticular abnormality and ground-glass opacities (2,3). Prone imaging confirms these possible abnormalities as true disease or not and can also facilitate detection of specific signs such as honeycombing in usual interstitial pneumonia (UIP) or subpleural sparing in nonspecific interstitial pneumonia (NSIP). As such, prone imaging may be omitted for cases where supine imaging does not demonstrate any potential subpleural abnormality. Similarly, prone imaging is not indicated in cases of fibrosis that are convincingly evident on supine imaging alone. As such, the need to include prone imaging or not should be part of any protocolling process performed by the radiologist or technologists prior to and during scan acquisition. In patients with established fibrosis, prone imaging should be omitted, and in patients where fibrosis has not yet been confirmed, prone imaging should be included. Some facilities review images at the scanner after inspiratory acquisition to determine the need for prone imaging; however, this is not practical at many facilities, and the determination must be made ahead of time.

Thin-section CT also plays a key role not just in initial diagnosis of fibrotic lung disease, but also in follow-up in cases of established lung fibrosis. Follow-up imaging can provide additional information on disease prognosis and progression, treatment efficacy, and disease complications. Follow-up imaging can be performed using a subjective (semiquantitative) or objective (quantitative) assessment method. In the subjective method, radiologists use a visual scoring system to grade the extent of the pulmonary fibrosis, which can then be correlated to clinical risk factors, including age, pretest probability, and pulmonary function tests, to assign a mortality risk assessment (13). While this subjective system has been found to be able to accurately predict patient mortality, due to the subjective nature of this grading method and its decreased sensitivity to changes in the setting of short-interval follow-up, there has been increasing research interest in developing an objective method for grading pulmonary fibrosis using computer-based CT analysis and machine learning. Researchers at National Jewish Health developed a process known as *data-driven textural analysis*, linking machine learning techniques (convolutional neural networks) with textural analysis to identify healthy and fibrotic lung at thin-section CT (14). The resulting data-driven textural analysis score can then be correlated to clinical risk factors, such as changes in pulmonary function. Rising data-driven textural analysis scores over time have been shown to be associated with increased disease progression and hospitalization.

### MRI Studies

MRI has limited value in the evaluation of ILD. MRI provides inadequate imaging of the lungs due to the relative lack of protons within the aerated lungs and air-induced artifacts. Additionally, the interstitium itself is at too small of a resolution to be visualized by available MRI techniques. Currently, thoracic MRI for evaluation of diffuse lung disease is limited to very specific pathologies for research purposes, such as cystic fibrosis and sarcoidosis (15,16).

## Thin-Section CT Search Pattern

Having an organized approach to thin-section CT is essential for interpretation of any possible diffuse lung disease, including IPF. Multiple possible search algorithms exist, none of which are proven superior; however, this guide suggests one such pattern as an example of a systematic approach to ensure identification of key findings. Each of the following should be evaluated: *(a)* the airways (including the trachea, main bronchi, and smaller bronchioles), *(b)* lung parenchyma (generally lobe by lobe), *(c)* pleura, *(d)* mediastinum, *(e)* upper abdomen, and *(e)* musculoskeletal system. As the primary concern of this guide is IPF, the majority of this review will focus on the lung parenchyma. However, the extra pulmonary findings can sometimes provide key diagnostic clues or prognostic information for the patient’s underlying disease, especially in the setting of connective tissue disease (CTD) (Table 1).

**Table 1:**
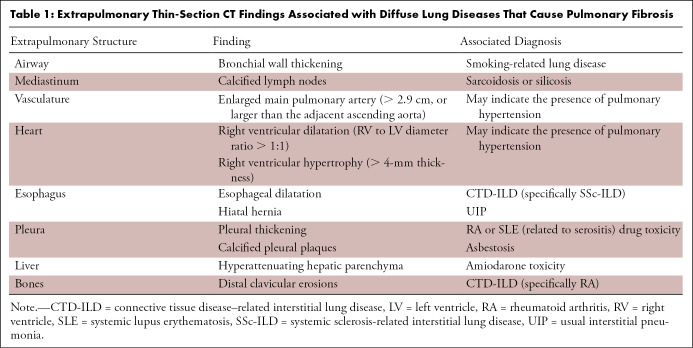
Extrapulmonary Thin-Section CT Findings Associated with Diffuse Lung Diseases That Cause Pulmonary Fibrosis

## Lung Parenchyma at Thin-Section CT

As already discussed, thin-section CT is the most sensitive radiologic examination to evaluate the lung parenchyma for evidence of ILD. The key anatomic components of the lung parenchyma that are evaluated for in IPF are the interstitium and secondary pulmonary lobule. The interstitium provides structural support to the lobule itself, and as such, diseases that distort the pulmonary interstitium result in distortion of the secondary pulmonary lobule. Although the axial interstitium surrounding the bronchovascular bundles is generally less important when evaluating for IPF, it may be important in other causes of pulmonary fibrosis. However, the peripheral interstitium and the intralobular interstitium are important in IPF. As such, when evaluating the lung parenchyma for IPF, abnormalities which predominantly affect these components lead to several key findings to evaluate (17). Their names and definitions are listed here with Table 2 summarizing these common findings and patterns and their clinical disease associations. Note that in practice, the clinical diseases discussed here can manifest with various imaging and histologic patterns and sometimes even multiple patterns in the same patient. For example, an imaging and histologic pattern of UIP may be ultimately diagnosed as CTD-related ILD rather than IPF due to the presence of systemic rheumatologic disease. As such, UIP refers specifically to the imaging or histologic pattern, while IPF refers to the clinical syndrome.

**Table 2:**
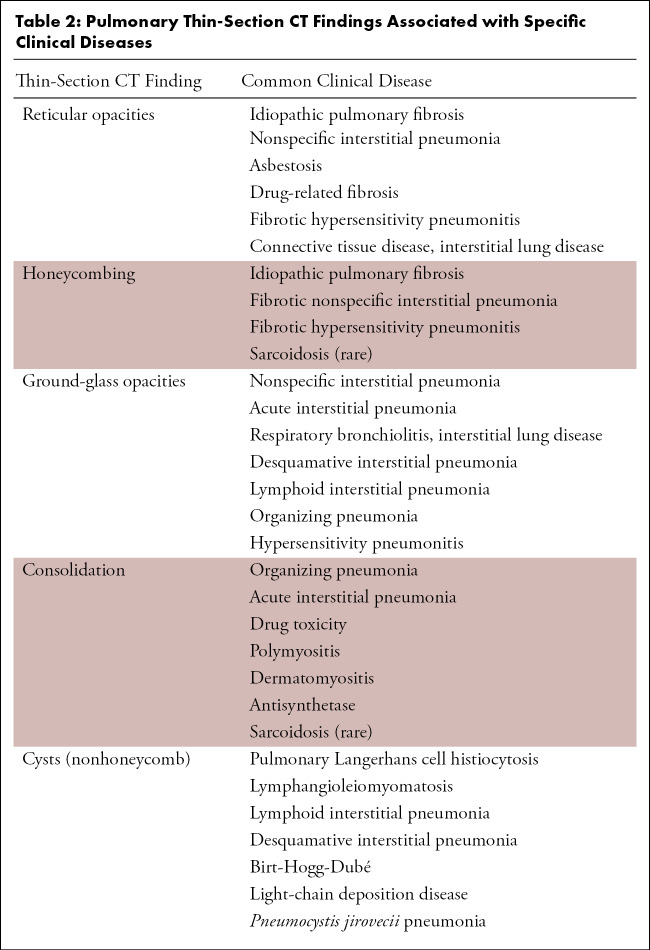
Pulmonary Thin-Section CT Findings Associated with Specific Clinical Diseases

## Key Definitions Seen in Patients with IPF

### Reticular Pattern

**Figure 2:**
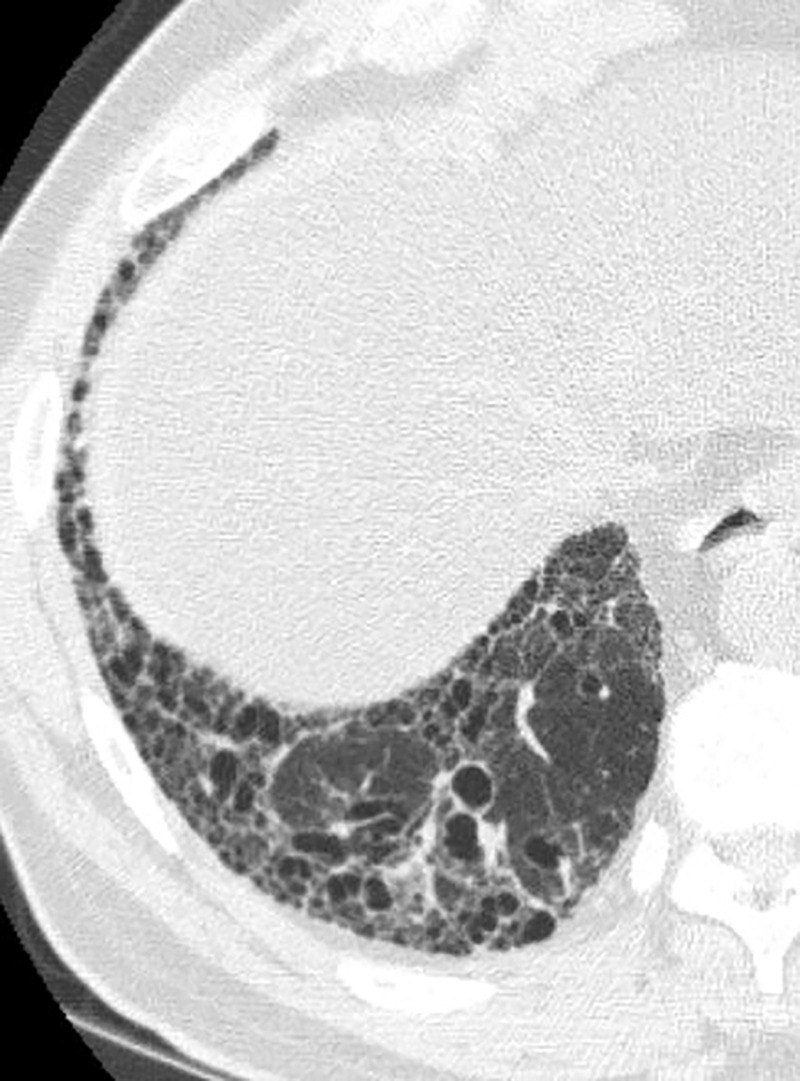
Reticular abnormality and architectural distortion. Reticular abnormality consists of a fine network or mesh of overlapping linear lines within the secondary pulmonary lobule. It is this network or mesh that separates reticular abnormality from ground-glass opacities, which are more homogeneous (see Figure 5). This case also shows associated architectural distortion with the normal secondary pulmonary lobules demonstrating tethering and warping of the normal hexagonal appearance. There is also mild honeycombing best seen in the costophrenic sulcus.

Reticular pattern, also sometimes referred to as *reticulation*, consists of a fine network or mesh of overlapping linear lines within the secondary pulmonary lobule (Fig 2). As such, this finding suggests an injury to the interstitium and is an indicator of fibrotic ILD in many cases. It is frequently seen in association with other evidence of fibrosis, including architectural distortion and bronchiectasis (17).

### Architectural Distortion

Architectural distortion refers to any distortion of the normal lung parenchymal anatomy (Fig 2). In the setting of ILD, this generally refers to an abnormal appearance of the secondary pulmonary lobule shape or size with evidence of volume loss (17).

### Traction Bronchiectasis and Bronchiolectasis

**Figure 3:**
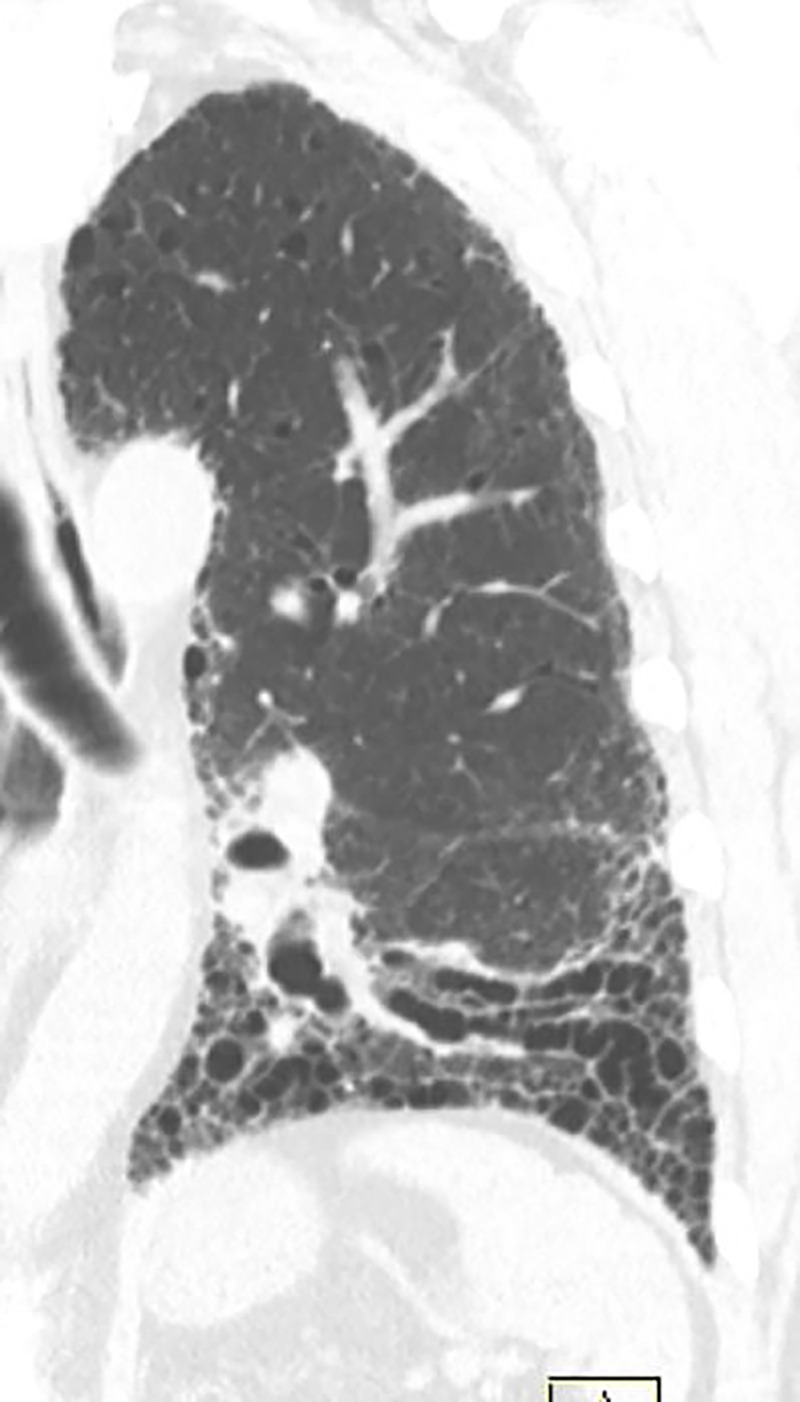
Traction bronchiectasis and bronchiolectasis. Zoomed view of coronal CT demonstrates continuity of dilated irregular airways with the more central bronchi (traction bronchiectasis and bronchiolectasis) in a patient with fibrotic nonspecific interstitial pneumonia.

Traction bronchiectasis and bronchiolectasis both refer to irreversible dilatation of an airway (Fig 3) related to surrounding or adjacent lung fibrosis. The dilated airway is often irregular and tortuous. Traction bronchiectasis is different from nontraction bronchiectasis, which is not associated with fibrosis and is usually associated with other signs of airways disease (17).

### Honeycomb Cysts

Honeycomb cysts are subpleural clustered cystic air spaces (Fig 4a). These are generally small in size (3–5 mm). To be considered as true honeycombing, the cysts must be contiguous, and must touch the pleural surface. They can be differentiated from emphysema on the basis of complete cyst walls being present in honeycombing. Honeycombing is also frequently multilayered, and it is important to distinguish honeycombing from traction bronchiolectasis. Traction bronchiectasis and bronchiolectasis both connect back to the more central airways, while honeycomb cysts should be peripheral true cysts without a discrete airway connection (17). Although it would seem that honeycombing would be an easy finding to identify, in practice, it can be challenging, and interobserver variability for presence or absence of honeycombing is borderline. However, as noted below in the categorization of UIP patterns, the presence of honeycombing is no longer considered a required feature in many cases of IPF in the appropriate clinical context. This alleviates some of the issues with the reliability of honeycombing identification in clinical practice.

**Figure 4a:**
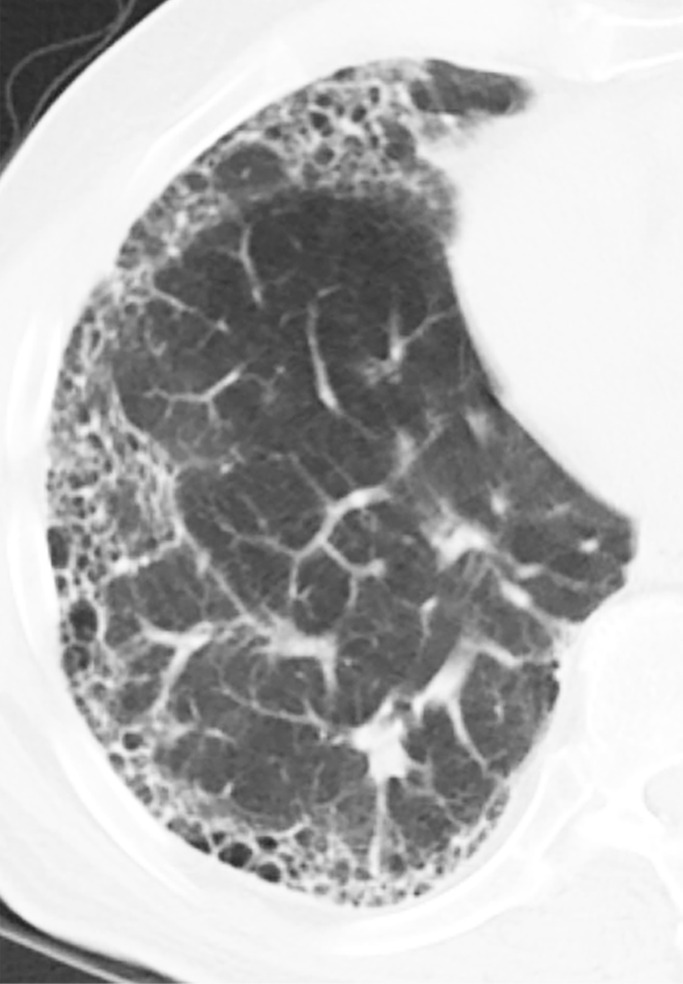
Honeycomb cysts and paraseptal emphysema. **(a)** Zoomed view of axial CT demonstrates classic honeycomb cyst formation with stacking subpleural cysts and associated adjacent fibrosis in a patient with idiopathic pulmonary fibrosis. **(b)** Zoomed view of axial CT in a different patient demonstrates paraseptal emphysema, characterized by a single layer of subpleural cysts, without any evidence of associated fibrosis in a patient with a history of marijuana cigarette smoking. The cysts of paraseptal emphysema are typically more than 1.0 cm in diameter, larger than honeycomb cysts, and have thinner walls.

**Figure 4b:**
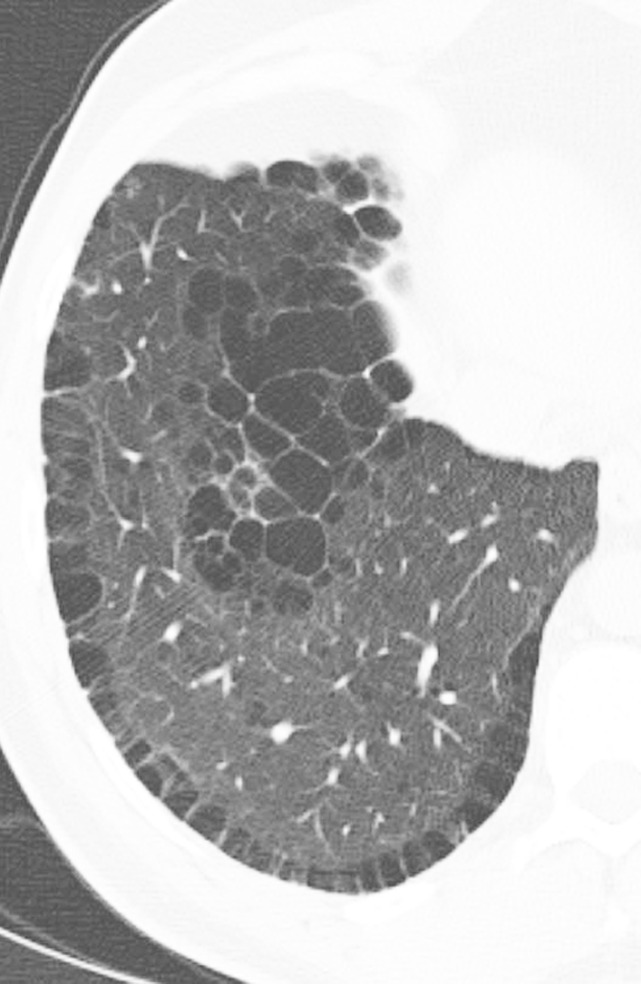
Honeycomb cysts and paraseptal emphysema. **(a)** Zoomed view of axial CT demonstrates classic honeycomb cyst formation with stacking subpleural cysts and associated adjacent fibrosis in a patient with idiopathic pulmonary fibrosis. **(b)** Zoomed view of axial CT in a different patient demonstrates paraseptal emphysema, characterized by a single layer of subpleural cysts, without any evidence of associated fibrosis in a patient with a history of marijuana cigarette smoking. The cysts of paraseptal emphysema are typically more than 1.0 cm in diameter, larger than honeycomb cysts, and have thinner walls.

### Basal Predominant

*Basal predominant* refers to the distribution of findings in the craniocaudal plane (superior to inferior) being more lower lung–predominant. This is the most common pattern observed in IPF. Other frequently used descriptors for disease distribution in the lungs include *upper lung–predominant*, *mid lung–predominant*, and *diffuse* (3).

## Key Definitions Seen in Patients without IPF

The following features may suggest an alternative diagnosis.

### Ground-Glass Opacity

**Figure 5:**
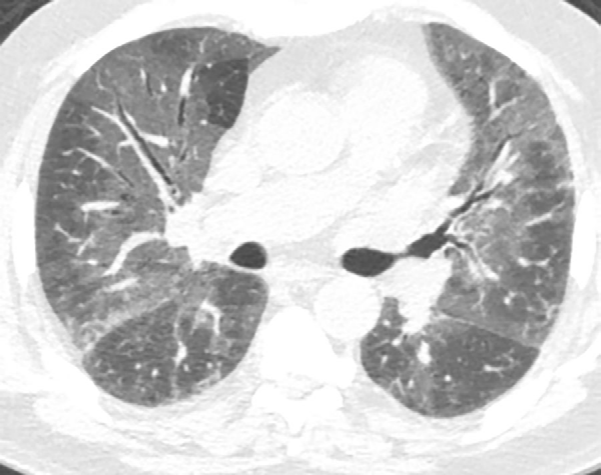
Ground-glass opacities. Axial CT image demonstrates homogeneous areas of increased lung attenuation in which the increased opacity does not obscure the underlying bronchial and vascular structures. This finding is not a feature typical of usual interstitial pneumonia or idiopathic pulmonary fibrosis but can be seen in the setting of overlapping or superimposed disease.

*Ground-glass opacity* refers to a homogeneous area of increased lung opacity (a process which partially fills the airspaces) in which the increased opacity does not obscure the underlying bronchial and vascular structures (Fig 5) (17). As such, it is less dense than consolidation (which completely fills the airspaces). A variety of pathologic mechanisms can result in ground-glass opacity. In cases related to pulmonary fibrosis, the ground-glass opacities may represent a very fine interstitial fibrosis that is beyond the spatial resolution of thin-section CT, rather than active lung inflammation. The presence of ground-glass opacities frequently represents a reversible disease process if it is not associated with other evidence of fibrosis, such as traction bronchiectasis. Abundant ground-glass opacities in cases of pulmonary fibrosis can also be due to acute exacerbation of ILD, superimposed infection, or pulmonary edema.

### Consolidation

**Figure 6:**
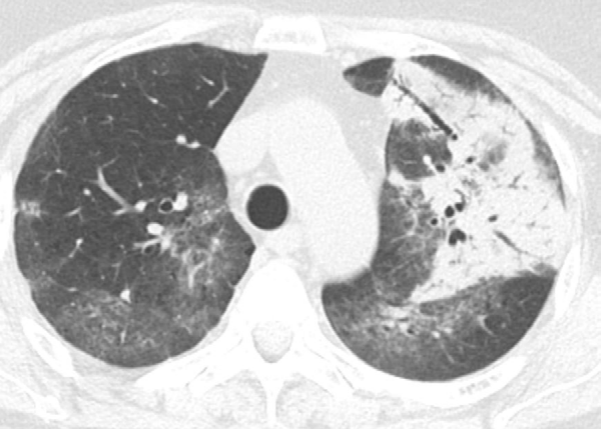
Consolidation. Left upper lobe consolidation in a patient with infectious pneumonia. The lung attenuation is diffusely increased, with air bronchograms, and the underlying vessels are completely obscured by the airspace opacity. Ground-glass opacities are seen in the nonconsolidated lung bilaterally. This finding is not a feature typical of usual interstitial pneumonia or idiopathic pulmonary fibrosis but can be seen in the setting of overlapping or superimposed disease.

*Consolidation* refers to an area of increased lung opacity (a process which completely fills the airspaces) in which the increased opacity does obscure the underlying vascular and bronchial structures (Fig 6) (17). Consolidation is not a feature of UIP but could be seen in patients with IPF with overlapping infection or malignancy.

### Micronodules

Micronodules are small rounded opacities, less than 3 mm in size, that can follow variable distributions within the lung parenchyma. A full distribution of nodular patterns is outside the scope of this work, but the presence of micronodules is important to note when classifying cases of possible IPF (17). Common diseases associated with micronodules and possible fibrosis include sarcoidosis, pneumoconiosis, and hypersensitivity pneumonitis (HP).

### Mosaic Attenuation

*Mosaic attenuation* refers to a pattern of heterogeneous attenuation of the lung parenchyma at thin-section CT with generally well-defined geographic borders corresponding to the outlines of pulmonary lobules (Fig 7). Causes of mosaic attenuation include both vascular disease (such as severe pulmonary hypertension or chronic thromboembolic disease) and small airways disease (such as HP, asthma, or constrictive bronchiolitis). Expiratory CT is very helpful in differentiating airway causes of mosaic attenuation (3,17).

**Figure 7a:**
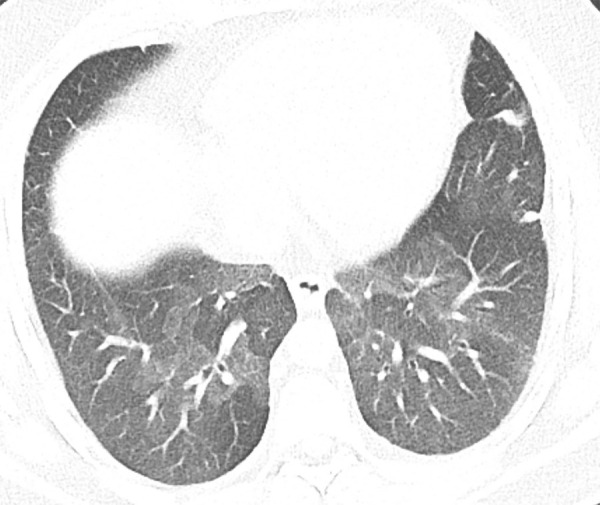
Mosaic attenuation and air trapping. **(a)** Axial inspiratory CT scan demonstrates mosaic attenuation due to ground-glass opacity in a case of respiratory bronchiolitis-associated interstitial lung disease. **(b)** Axial inspiratory CT scan demonstrates mosaic attenuation due to small airways disease in a case of constrictive bronchiolitis. **(c)** Axial expiratory CT imaging (same patient as **b**) demonstrates accentuation of the areas of hyperlucency from the adjacent lung, confirming small airways disease as the cause of mosaic attenuation.

**Figure 7b:**
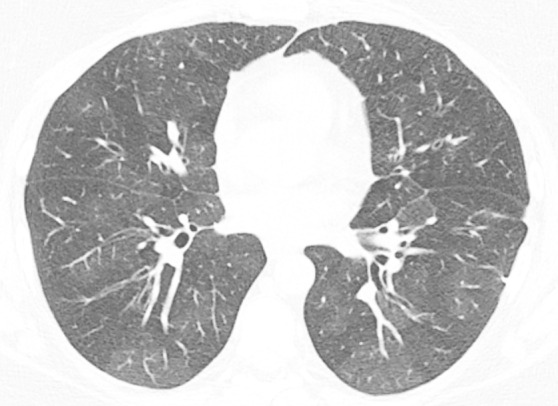
Mosaic attenuation and air trapping. **(a)** Axial inspiratory CT scan demonstrates mosaic attenuation due to ground-glass opacity in a case of respiratory bronchiolitis-associated interstitial lung disease. **(b)** Axial inspiratory CT scan demonstrates mosaic attenuation due to small airways disease in a case of constrictive bronchiolitis. **(c)** Axial expiratory CT imaging (same patient as **b**) demonstrates accentuation of the areas of hyperlucency from the adjacent lung, confirming small airways disease as the cause of mosaic attenuation.

**Figure 7c:**
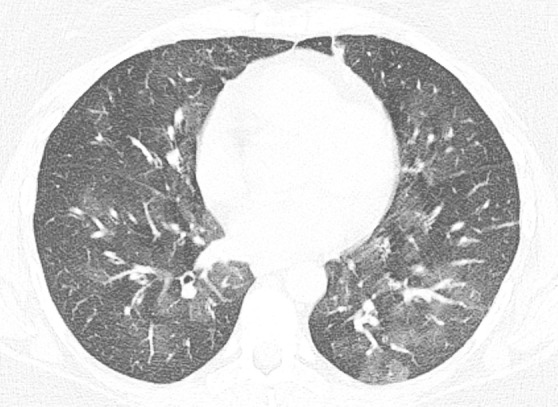
Mosaic attenuation and air trapping. **(a)** Axial inspiratory CT scan demonstrates mosaic attenuation due to ground-glass opacity in a case of respiratory bronchiolitis-associated interstitial lung disease. **(b)** Axial inspiratory CT scan demonstrates mosaic attenuation due to small airways disease in a case of constrictive bronchiolitis. **(c)** Axial expiratory CT imaging (same patient as **b**) demonstrates accentuation of the areas of hyperlucency from the adjacent lung, confirming small airways disease as the cause of mosaic attenuation.

### Air Trapping

*Air trapping* refers to a small airway disease resulting in areas of lucency with hyperinflation at expiratory CT (Fig 7). Mild cases of air trapping may only be identifiable on expiratory scans, but more severe cases may show evidence of mosaic attenuation at inspiratory CT (17). Air trapping in the setting of ILD is most commonly seen associated with HP, constrictive bronchiolitis, and sarcoidosis.

### Cysts

Cysts, by definition, have discrete walls. Cysts that are not honeycomb cysts (ie, do not stack along the subpleural lung) suggest a diagnosis other than IPF. Cyst appearance and distribution can be quite useful for narrowing the differential diagnosis for diffuse lung diseases; however, this is beyond the scope of this guide (17). Lung cysts can be seen in several diffuse lung diseases including pulmonary Langerhans cell histiocytosis, lymphangioleiomyomatosis, lymphoid interstitial pneumonia, desquamative interstitial pneumonia, Birt-Hogg-Dubé, light-chain deposition disease, and *Pneumocystis jiroveccii* pneumonia.

## Confounding Imaging Features

### Honeycomb Cysts versus Paraseptal Emphysema

On thin-section CT scans, honeycombing appears as subpleural cystic air spaces, usually 5 mm or less in diameter, though sometimes as large as 1–2 cm, with well-defined walls. Honeycomb cysts typically share walls and occur in multiple layers, though early honeycombing may manifest as a single layer of subpleural cysts (18).

Paraseptal emphysema appears as subpleural cysts or lucencies that can mimic honeycombing. However, paraseptal emphysema usually occurs in a single subpleural layer, with cyst size usually larger than 1 cm, and is often associated with centrilobular emphysema. Additionally, paraseptal emphysema generally has thinner walls than honeycomb cysts and also lacks the architectural distortion and reticular abnormality seen in association with pulmonary fibrosis and honeycomb cyst formation (Fig 4) (18).

### Honeycomb Cysts versus Traction Bronchiectasis and Bronchiolectasis

Traction bronchiectasis and bronchiolectasis can generally be separated from honeycombing by carefully examining contiguous thin-section CT sections and observing continuity and branching of bronchi and bronchioles rather than discrete honeycomb cysts (Fig 3). In some patients, evaluating sagittal and coronal reformations may help demonstrate characteristic airway branching better than axial images (18).

### Traction Bronchiectasis versus Bronchiectasis from Other Causes

Traction bronchiectasis and bronchiectasis due to chronic inflammation and intrinsic airways disease should be distinguished. Traction bronchiectasis refers to airway dilatation caused by an underlying pulmonary fibrotic parenchymal abnormality that causes the bronchiectasis rather than an airway-centered process, such as chronic bronchitis, that leads to airway dilatation. Traction bronchiectasis generally lacks clinically significant wall thickening or mucoid impaction, and it is always adjacent to other findings of fibrosis, such as irregular reticular opacities, subtle ground-glass opacities, honeycombing, architectural distortion, and volume loss, which result in a “tethering” effect that causes the bronchiectasis (Fig 8) (18).

**Figure 8a:**
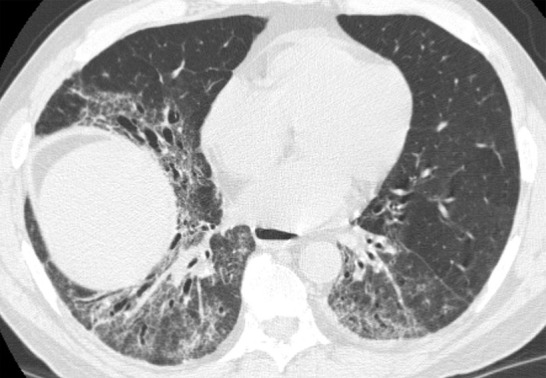
Comparison of traction bronchiectasis with bronchiectasis from chronic airway inflammation. **(a)** Axial CT scan demonstrates traction bronchiectasis in a patient with fibrotic nonspecific interstitial pneumonia. Note the substantial reticular abnormality and architectural distortion adjacent to the irregular dilated airways. **(b)** Axial CT scan demonstrates cylindrical bronchiectasis in the right lower lobe of a patient with underlying immunodeficiency. In contrast to traction bronchiectasis, this postinflammatory bronchiectasis lacks adjacent fibrosis and “tethering” of the airways. There are also associated findings of bronchial wall thickening and mucoid impaction.

**Figure 8b:**
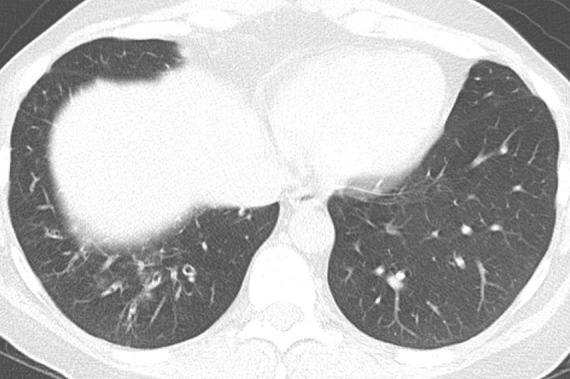
Comparison of traction bronchiectasis with bronchiectasis from chronic airway inflammation. **(a)** Axial CT scan demonstrates traction bronchiectasis in a patient with fibrotic nonspecific interstitial pneumonia. Note the substantial reticular abnormality and architectural distortion adjacent to the irregular dilated airways. **(b)** Axial CT scan demonstrates cylindrical bronchiectasis in the right lower lobe of a patient with underlying immunodeficiency. In contrast to traction bronchiectasis, this postinflammatory bronchiectasis lacks adjacent fibrosis and “tethering” of the airways. There are also associated findings of bronchial wall thickening and mucoid impaction.

## Imaging Findings of IPF and the UIP Pattern

A UIP pattern on CT scan or histologic evaluation is the morphologic hallmark of IPF. However, a UIP pattern may also be found in several other conditions, including chronic HP, drug toxicity, asbestosis, and CTDs such as rheumatoid arthritis (3,6). There is also a category known as *familial fibrosis* (fibrosis that has a genetic component including short telomeres and/or tends to run in families) which can manifest with a UIP pattern (19). All patients with lung fibrosis require detailed clinical evaluation for evaluation of potential underlying cause (3,6).

Many cases of UIP have typical CT appearances, strongly supporting the diagnosis. However, there is increasing recognition that the appearance of UIP may be nontypical, and diagnostic guidelines have been expanded to recognize features that permit the diagnosis of IPF in less typical cases (3,6).

## Diagnostic Categories of Pulmonary Fibrosis at Thin-Section CT

Two international multidisciplinary groups have published recent statements on the diagnosis of IPF (see Table 3): the Fleischner Society and a joint working group formed by the American Thoracic Society, European Respiratory Society, Japanese Respiratory Society, and Latin American Thoracic Society (ATS/ERS/JRS/ALAT) (3,6). Each group defined four diagnostic categories of pulmonary fibrosis at thin-section CT in patients suspected of having UIP or IPF, listed below. These categories are defined by the presence or absence of the specific defined features. The following is the Fleischner Society criteria.

**Table 3:**
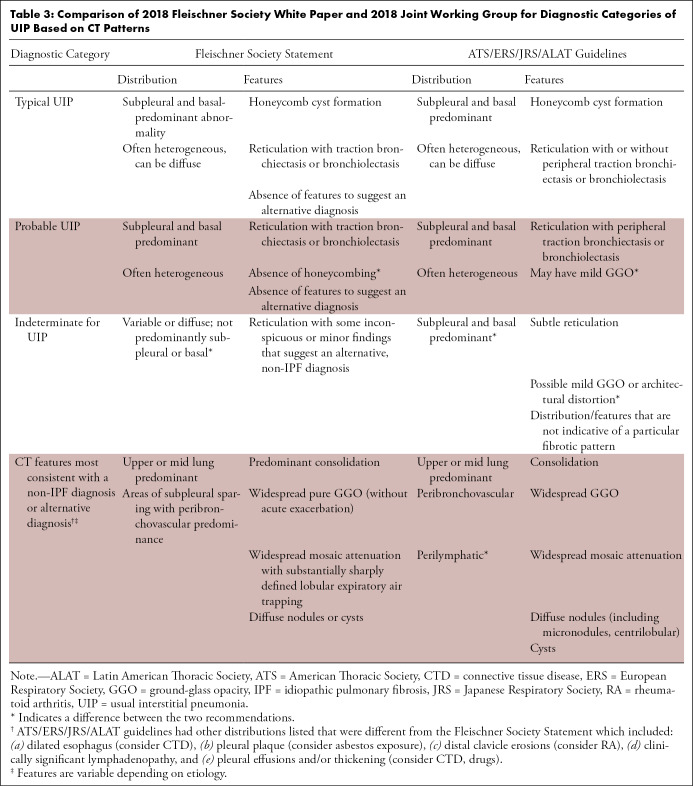
Comparison of 2018 Fleischner Society White Paper and 2018 Joint Working Group for Diagnostic Categories of UIP Based on CT Patterns

### Typical UIP Pattern

This is the classic pattern and has a predictive value of greater than 90% for histologic UIP (example shown in Fig 9). Must have the following features:

1. Subpleural and basal-predominant reticular abnormality (frequently spatially heterogeneous) with traction bronchiectasis or bronchiolectasis.
2. Honeycomb cyst formation.
3. Absence of features to suggest an alternative diagnosis.

**Figure 9a:**
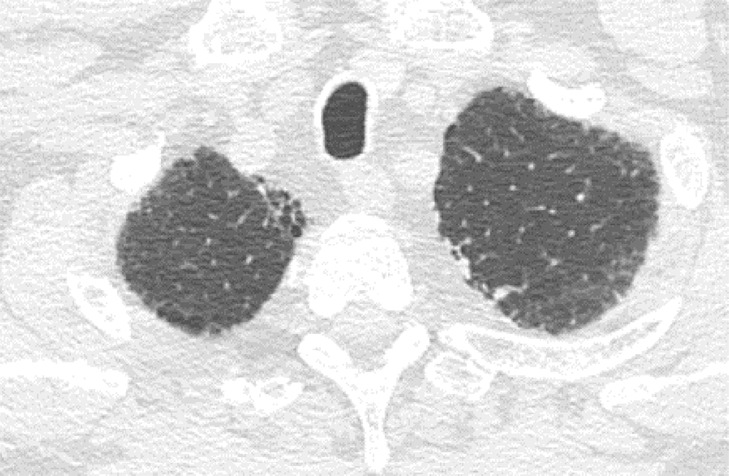
Usual interstitial pneumonia pattern. **(a–d)** Axial inspiratory CT images demonstrate peripheral and basilar-predominant reticular abnormality, with associated traction bronchiectasis and honeycombing. This is a usual interstitial pneumonia pattern based on Fleischner and American Thoracic Society guidelines. **(e)** Coronal inspiratory CT scan confirms the lower lung–predominant distribution of disease in the craniocaudal plane.

**Figure 9b:**
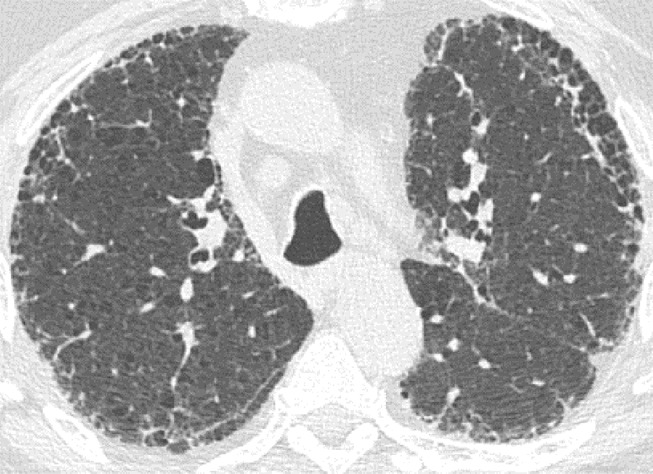
Usual interstitial pneumonia pattern. **(a–d)** Axial inspiratory CT images demonstrate peripheral and basilar-predominant reticular abnormality, with associated traction bronchiectasis and honeycombing. This is a usual interstitial pneumonia pattern based on Fleischner and American Thoracic Society guidelines. **(e)** Coronal inspiratory CT scan confirms the lower lung–predominant distribution of disease in the craniocaudal plane.

**Figure 9c:**
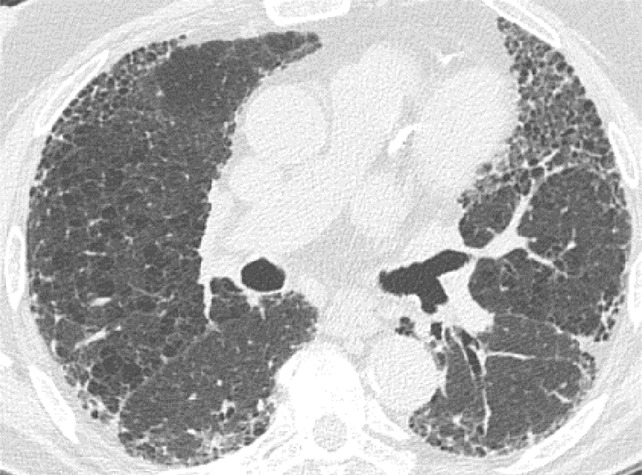
Usual interstitial pneumonia pattern. **(a–d)** Axial inspiratory CT images demonstrate peripheral and basilar-predominant reticular abnormality, with associated traction bronchiectasis and honeycombing. This is a usual interstitial pneumonia pattern based on Fleischner and American Thoracic Society guidelines. **(e)** Coronal inspiratory CT scan confirms the lower lung–predominant distribution of disease in the craniocaudal plane.

**Figure 9d:**
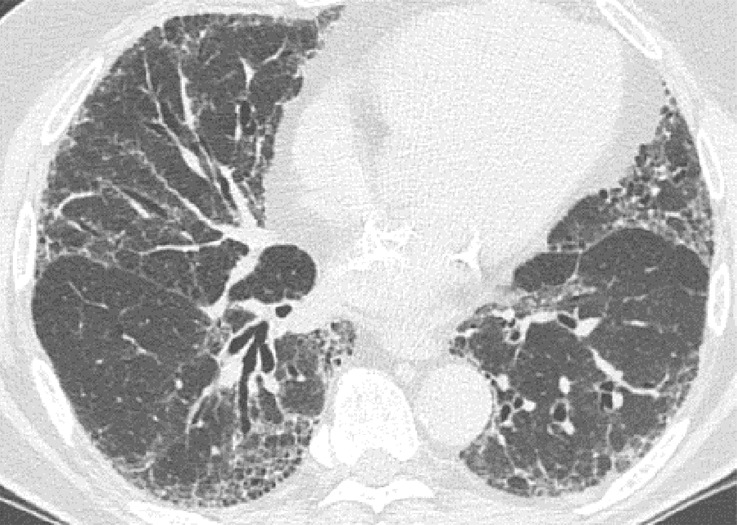
Usual interstitial pneumonia pattern. **(a–d)** Axial inspiratory CT images demonstrate peripheral and basilar-predominant reticular abnormality, with associated traction bronchiectasis and honeycombing. This is a usual interstitial pneumonia pattern based on Fleischner and American Thoracic Society guidelines. **(e)** Coronal inspiratory CT scan confirms the lower lung–predominant distribution of disease in the craniocaudal plane.

**Figure 9e:**
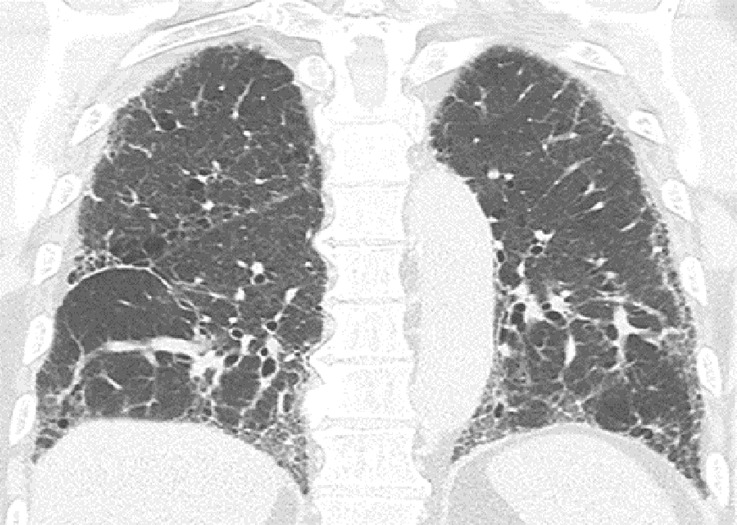
Usual interstitial pneumonia pattern. **(a–d)** Axial inspiratory CT images demonstrate peripheral and basilar-predominant reticular abnormality, with associated traction bronchiectasis and honeycombing. This is a usual interstitial pneumonia pattern based on Fleischner and American Thoracic Society guidelines. **(e)** Coronal inspiratory CT scan confirms the lower lung–predominant distribution of disease in the craniocaudal plane.

### Probable UIP Pattern

This pattern was previously known as *possible* UIP pattern. Probable UIP pattern is similar to the typical UIP pattern, except that honeycombing is not present. About 80% of patients with this pattern have UIP (example shown in Fig 10). Must have the following features:

1. Subpleural and basal-predominant reticular abnormality (frequently heterogeneous) with traction bronchiectasis and bronchiolectasis.
2. Absence of features to suggest an alternative diagnosis.

**Figure 10a:**
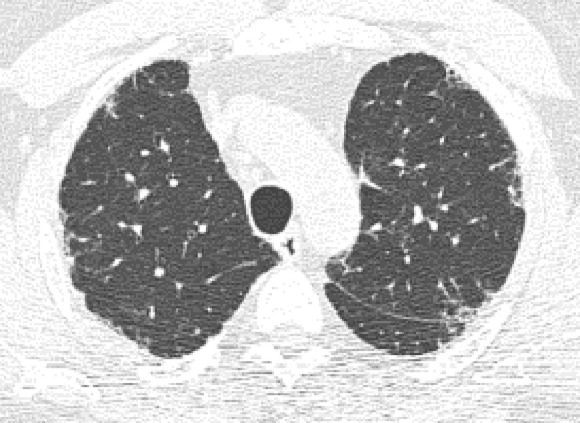
Probable usual interstitial pneumonia pattern. **(a–c)** Axial inspiratory CT images demonstrate peripheral and basilar-predominant reticular abnormality, with mild peripheral traction bronchiectasis or bronchiolectasis, but without associated honeycombing, making this a probable UIP pattern based on Fleischner and American Thoracic Society guidelines. **(d)** Coronal inspiratory CT scan confirms the lower lung–predominant distribution of disease in the craniocaudal plane.

**Figure 10b:**
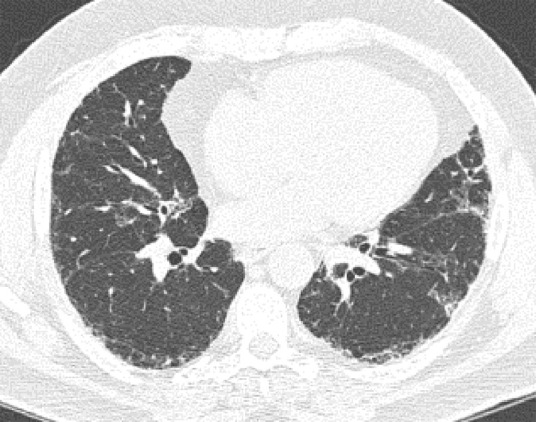
Probable usual interstitial pneumonia pattern. **(a–c)** Axial inspiratory CT images demonstrate peripheral and basilar-predominant reticular abnormality, with mild peripheral traction bronchiectasis or bronchiolectasis, but without associated honeycombing, making this a probable UIP pattern based on Fleischner and American Thoracic Society guidelines. **(d)** Coronal inspiratory CT scan confirms the lower lung–predominant distribution of disease in the craniocaudal plane.

**Figure 10c:**
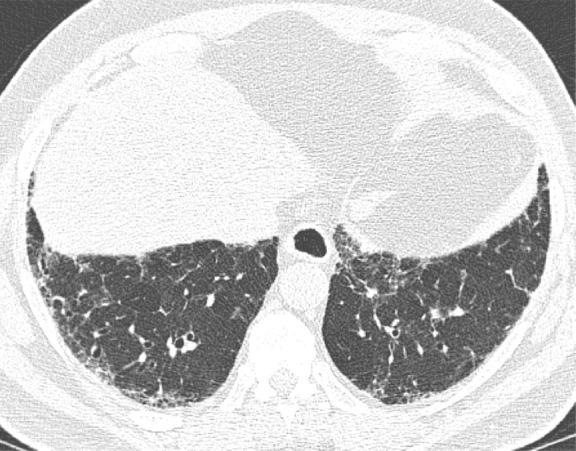
Probable usual interstitial pneumonia pattern. **(a–c)** Axial inspiratory CT images demonstrate peripheral and basilar-predominant reticular abnormality, with mild peripheral traction bronchiectasis or bronchiolectasis, but without associated honeycombing, making this a probable UIP pattern based on Fleischner and American Thoracic Society guidelines. **(d)** Coronal inspiratory CT scan confirms the lower lung–predominant distribution of disease in the craniocaudal plane.

**Figure 10d:**
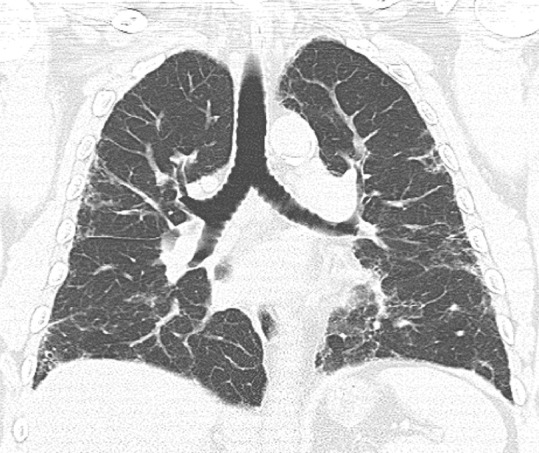
Probable usual interstitial pneumonia pattern. **(a–c)** Axial inspiratory CT images demonstrate peripheral and basilar-predominant reticular abnormality, with mild peripheral traction bronchiectasis or bronchiolectasis, but without associated honeycombing, making this a probable UIP pattern based on Fleischner and American Thoracic Society guidelines. **(d)** Coronal inspiratory CT scan confirms the lower lung–predominant distribution of disease in the craniocaudal plane.

### Indeterminate for UIP Pattern

In this pattern, the CT features are not sufficient for a firm diagnosis of UIP. About 50% of cases with this pattern have UIP (example shown in Fig 11). Should have the following features:

1. Distribution of reticular abnormality not predominantly subpleural or not predominantly basal.
2. May have some inconspicuous or minor findings that suggest an alternative, non-IPF diagnosis (as defined below). This would include a small amount of ground-glass opacity or a minor degree of air trapping seen in several lobules.

**Figure 11a:**
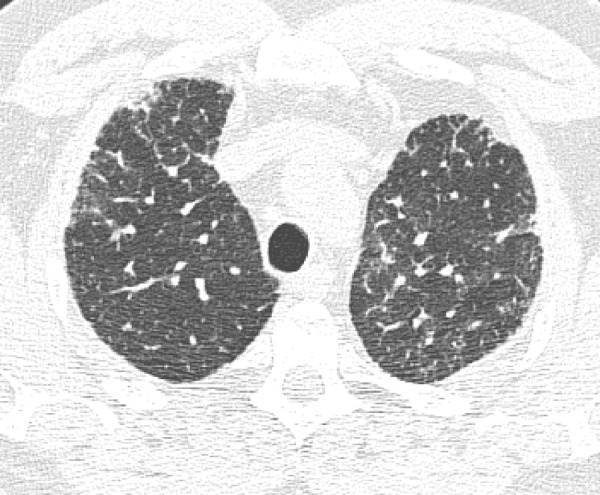
CT pattern indeterminate for usual interstitial pneumonia. **(a–c)** Axial inspiratory CT images demonstrate peripheral and lower lung–predominant reticular abnormality with architectural distortion, including traction bronchiectasis. However, there is slightly more ground-glass abnormality than typically seen with a confident diagnosis of a usual interstitial pneumonia pattern, and the reticular abnormality extends along the bronchovascular bundles rather than being confined to the subpleural lung.

**Figure 11b:**
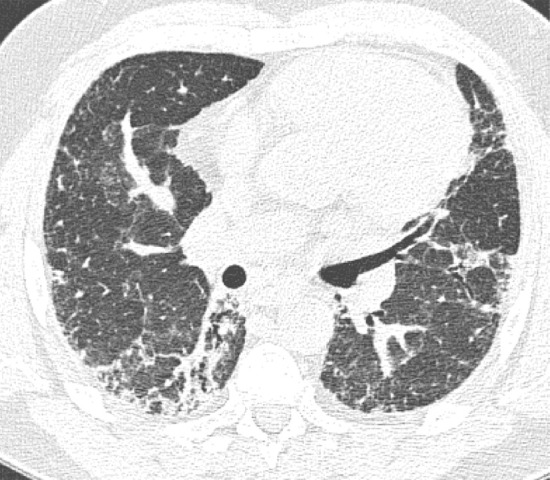
CT pattern indeterminate for usual interstitial pneumonia. **(a–c)** Axial inspiratory CT images demonstrate peripheral and lower lung–predominant reticular abnormality with architectural distortion, including traction bronchiectasis. However, there is slightly more ground-glass abnormality than typically seen with a confident diagnosis of a usual interstitial pneumonia pattern, and the reticular abnormality extends along the bronchovascular bundles rather than being confined to the subpleural lung.

**Figure 11c:**
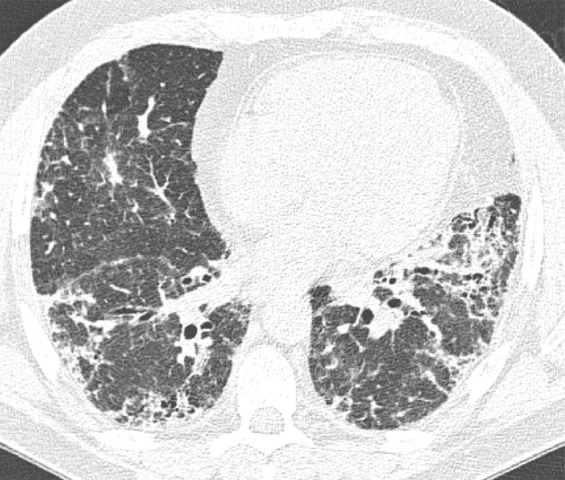
CT pattern indeterminate for usual interstitial pneumonia. **(a–c)** Axial inspiratory CT images demonstrate peripheral and lower lung–predominant reticular abnormality with architectural distortion, including traction bronchiectasis. However, there is slightly more ground-glass abnormality than typically seen with a confident diagnosis of a usual interstitial pneumonia pattern, and the reticular abnormality extends along the bronchovascular bundles rather than being confined to the subpleural lung.

If an indeterminate pattern is identified, an appropriate differential diagnosis should be provided in the radiologic report (for example: “Fibrosing interstitial pneumonia with CT features indeterminate for UIP. Differential diagnosis might include fibrotic HP, NSIP, or UIP”).

### CT Features Most Consistent with a Non-IPF Diagnosis

**Figure 12:**
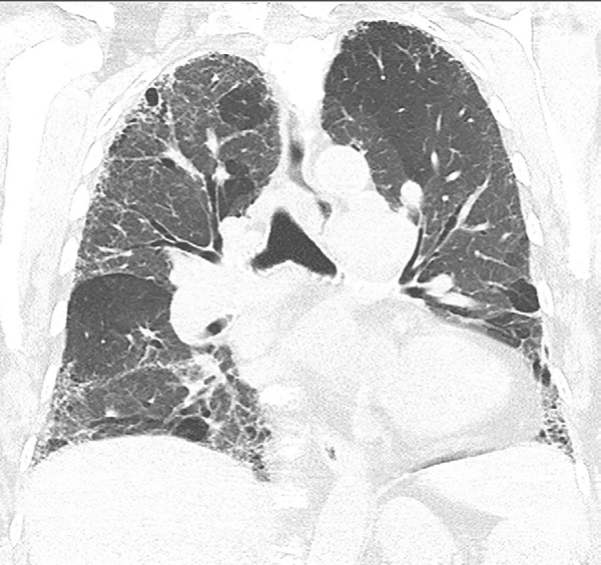
CT pattern most consistent with a nonidiopathic pulmonary fibrosis diagnosis. Coronal CT scan demonstrates pulmonary fibrosis but with substantial upper lung reticular opacities and architectural distortion in addition to ground-glass abnormality and mosaic attenuation. This combination points to chronic hypersensitivity pneumonitis as the likely diagnosis rather than idiopathic pulmonary fibrosis.

With this pattern, an alternative diagnosis should be strongly suggested. However, about 50% of cases with this pattern ultimately have UIP on histologic examination findings and a clinical diagnosis of IPF (example shown in Fig 12). This category specifically refers to a non-IPF diagnosis rather than a non-UIP diagnosis to separate the clinical disease (IPF) from the imaging and histologic patterns (UIP). Any of the following features:

1. Upper- or midlung-predominant disease distribution (suggests HP).
2. Areas of subpleural sparing with a peribronchovascular distribution (suggests NSIP, may be better seen on prone imaging).
3. Significant findings typically found with non-IPF diagnosis (predominant consolidation, extensive pure ground-glass opacity [without acute exacerbation], extensive mosaic attenuation with extensive sharply defined lobular expiratory air trapping [suggests HP], diffuse nodules or cysts: see above definitions).

In radiologic reports, the specific alternative diagnosis (or differential diagnosis) should be provided (for example: “CT features most consistent with fibrotic HP. However, the differential diagnosis might also include NSIP or atypical UIP*”*).

It is useful to remember that the only difference between the typical UIP pattern and probable UIP pattern is the presence of honeycomb cysts in the typical UIP pattern. It is similarly useful to remember that the 2011 ATS guidelines used the term *possible* UIP pattern, which is now renamed to probable UIP pattern, as the majority of these cases would have a pathologic pattern of probable or definite UIP at surgical lung biopsy (82%–94%) (3,6).

## Differences between the Fleischner Society Statement and the ATS/ERS/JRS/ALAT IPF Guidelines

Both the ATS/ERS/JRS/ALAT guidelines and Fleischner statement (3,6) are evidence based (ATS/ERS/JRS/ALAT guidelines are clinical practice guidelines that used Grading of Recommendations Assessment, Development and Evaluation methods, while Fleischner is expert consensus, but used a systematic literature search based on key questions). From an imaging standpoint, the categories of fibrotic lung disease based on thin-section CT evaluation are practically identical with only minor variation in nomenclature (see Table 3). However, there are some differences between the guideline statements with regard to diagnostic management. The most notable difference involves those patients with an unknown cause of disease and a probable UIP pattern on thin-section CT scans. In these cases, the Fleischner Society recommends no biopsy or bronchoalveolar lavage if the clinical context is appropriate for IPF (age > 60 years, no evidence of CTD or relevant exposure) and the ATS/ERS/JRS/ALAT suggests surgical biopsy and bronchoalveolar lavage in this population (conditional recommendation). Both statements recommend biopsy if the CT diagnosis is indeterminate or suggestive of an alternative diagnosis. Another difference is that the Fleischner statement recognizes the concept of a “working” or “provisional” diagnosis of IPF, discussed above, but the ATS/ERS/JRS/ALAT does not address this issue.

## Common Alternative Diagnoses to IPF

### NSIP

The thin-section CT abnormalities of NSIP can overlap with UIP but are frequently visually distinct. The predominant abnormalities feature more ground-glass opacities with frequent underlying reticular abnormality and traction bronchiectasis (Fig 12). Honeycombing can be seen with NSIP but is usually mild and should not be the predominant finding (in which cases, UIP should be considered). Although these features are generally lower lung predominant (similar to UIP), the distribution of ground-glass opacities frequently demonstrates a degree of subpleural sparing (unlike UIP). Furthermore, the traction bronchiectasis may demonstrate a patchier and more peribronchovascular distribution than that seen in UIP. NSIP often demonstrates gradual progression of fibrosis at thin-section CT, although the overall thin-section CT pattern still resembles NSIP. However, in a small number of patients, the fibrosis progresses so that the follow-up thin-section CT assumes more of a UIP pattern (Fig 13). As such, if the patient is imaged at an institution that does not have access to their prior imaging, the patient may be misdiagnosed as having a UIP or probable UIP pattern. Having prior imaging is therefore important to properly categorizing these patients (20).

**Figure 13a:**
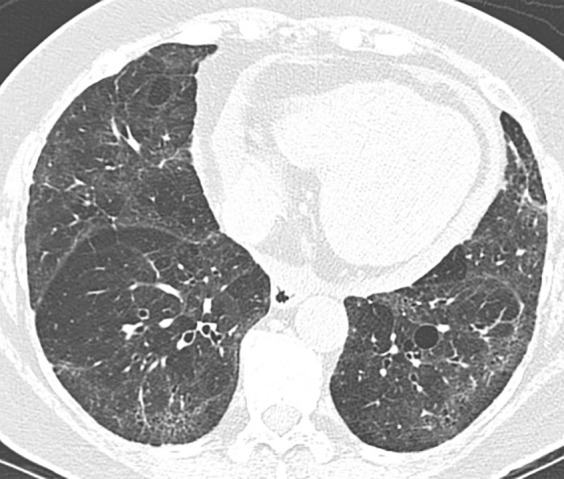
Nonspecific interstitial pneumonia. **(a)** Axial CT scan demonstrates fibrotic nonspecific interstitial pneumonia early in the disease course with lower lung fibrosis and ground-glass opacity with some subpleural sparing. **(b)** Axial CT scan 10 years later demonstrates substantial progression of the pulmonary fibrosis, which now demonstrates increased reticular abnormality, less ground-glass abnormality, and more traction bronchiectasis. Understanding the evolution of the disease by reviewing the prior imaging is critical in these cases.

**Figure 13b:**
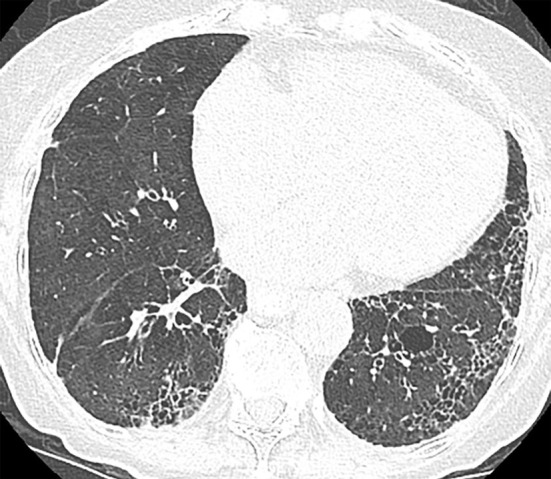
Nonspecific interstitial pneumonia. **(a)** Axial CT scan demonstrates fibrotic nonspecific interstitial pneumonia early in the disease course with lower lung fibrosis and ground-glass opacity with some subpleural sparing. **(b)** Axial CT scan 10 years later demonstrates substantial progression of the pulmonary fibrosis, which now demonstrates increased reticular abnormality, less ground-glass abnormality, and more traction bronchiectasis. Understanding the evolution of the disease by reviewing the prior imaging is critical in these cases.

### Fibrotic Hypersensitivity Pneumonitis

Although classically thought of as upper lung predominant, the thin-section CT abnormalities of fibrotic HP can mimic UIP (including lower lung predominance) in upwards of 50% of cases (Fig 14). However, some features, such as air trapping (particularly lobular air trapping) and centrilobular ground-glass nodules, are much more commonly seen in HP than UIP, and their presence should suggest an alternative diagnosis (21).

**Figure 14a:**
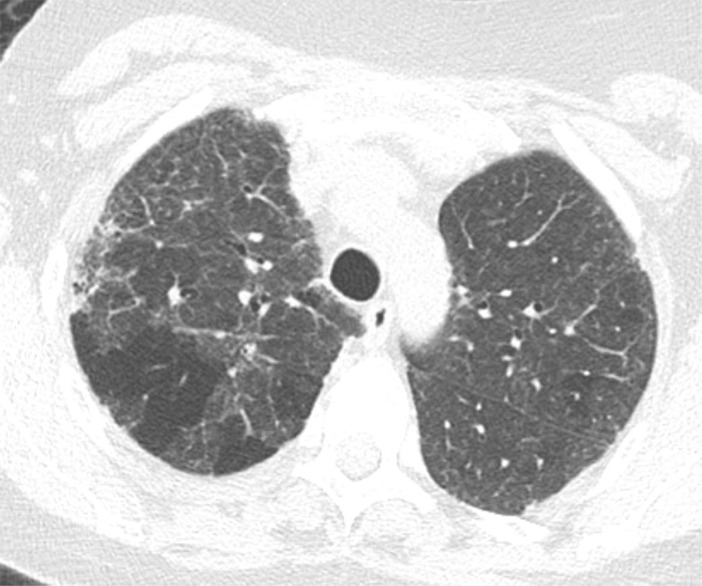
Fibrotic hypersensitivity pneumonitis. **(a–c)** Axial inspiratory CT scans demonstrate substantial ground-glass abnormality, mild centrilobular nodularity, and moderate mosaic attenuation, in addition to peribronchovascular and lower lung–predominant fibrosis. **(d–f)** Axial expiratory CT images confirm air trapping as the cause of mosaic attenuation. The degree of ground-glass abnormality, the degree of air trapping, and the peribronchovascular predominance of fibrosis would result in this case being categorized as most consistent with a nonidiopathic pulmonary fibrosis diagnosis.

**Figure 14b:**
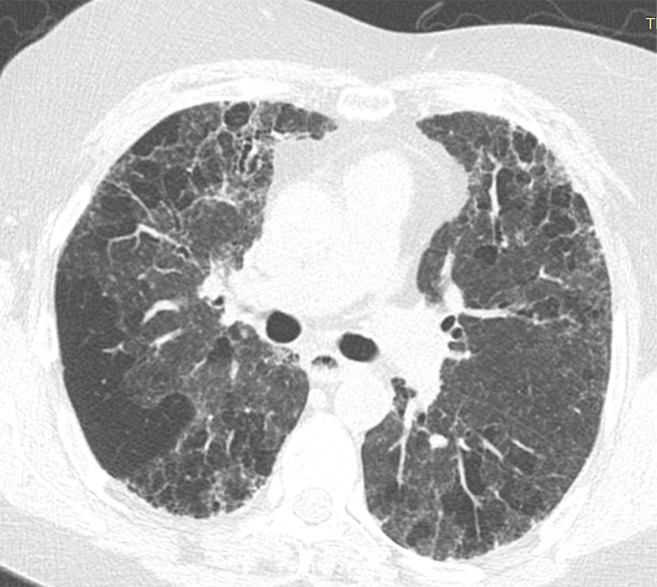
Fibrotic hypersensitivity pneumonitis. **(a–c)** Axial inspiratory CT scans demonstrate substantial ground-glass abnormality, mild centrilobular nodularity, and moderate mosaic attenuation, in addition to peribronchovascular and lower lung–predominant fibrosis. **(d–f)** Axial expiratory CT images confirm air trapping as the cause of mosaic attenuation. The degree of ground-glass abnormality, the degree of air trapping, and the peribronchovascular predominance of fibrosis would result in this case being categorized as most consistent with a nonidiopathic pulmonary fibrosis diagnosis.

**Figure 14c:**
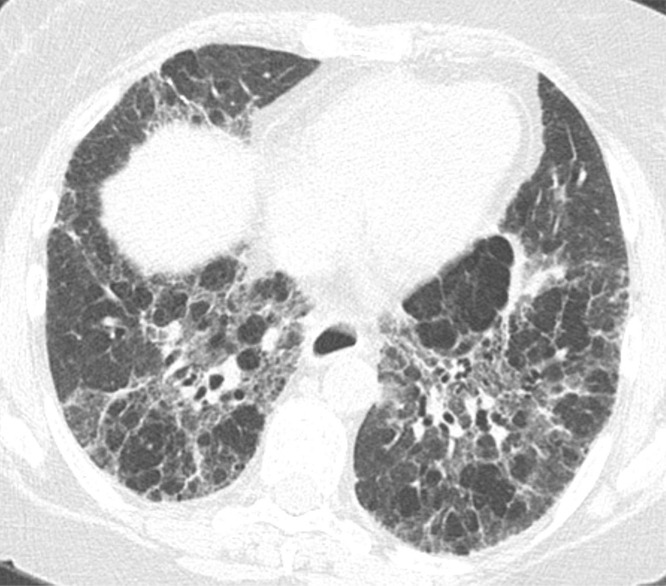
Fibrotic hypersensitivity pneumonitis. **(a–c)** Axial inspiratory CT scans demonstrate substantial ground-glass abnormality, mild centrilobular nodularity, and moderate mosaic attenuation, in addition to peribronchovascular and lower lung–predominant fibrosis. **(d–f)** Axial expiratory CT images confirm air trapping as the cause of mosaic attenuation. The degree of ground-glass abnormality, the degree of air trapping, and the peribronchovascular predominance of fibrosis would result in this case being categorized as most consistent with a nonidiopathic pulmonary fibrosis diagnosis.

**Figure 14d:**
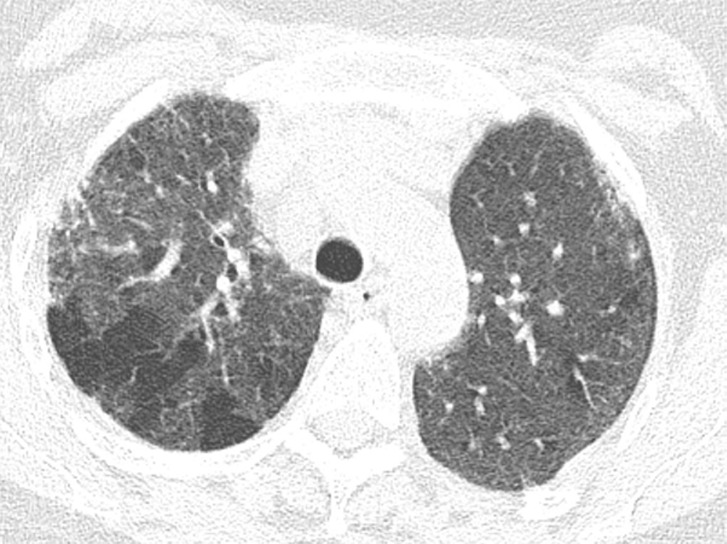
Fibrotic hypersensitivity pneumonitis. **(a–c)** Axial inspiratory CT scans demonstrate substantial ground-glass abnormality, mild centrilobular nodularity, and moderate mosaic attenuation, in addition to peribronchovascular and lower lung–predominant fibrosis. **(d–f)** Axial expiratory CT images confirm air trapping as the cause of mosaic attenuation. The degree of ground-glass abnormality, the degree of air trapping, and the peribronchovascular predominance of fibrosis would result in this case being categorized as most consistent with a nonidiopathic pulmonary fibrosis diagnosis.

**Figure 14e:**
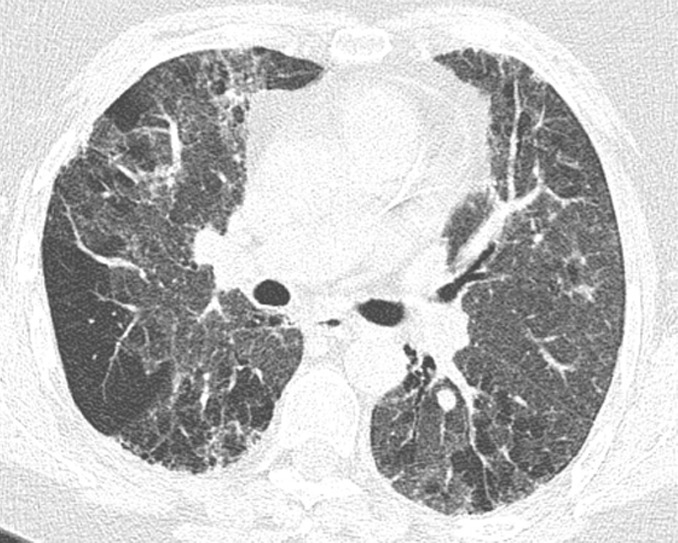
Fibrotic hypersensitivity pneumonitis. **(a–c)** Axial inspiratory CT scans demonstrate substantial ground-glass abnormality, mild centrilobular nodularity, and moderate mosaic attenuation, in addition to peribronchovascular and lower lung–predominant fibrosis. **(d–f)** Axial expiratory CT images confirm air trapping as the cause of mosaic attenuation. The degree of ground-glass abnormality, the degree of air trapping, and the peribronchovascular predominance of fibrosis would result in this case being categorized as most consistent with a nonidiopathic pulmonary fibrosis diagnosis.

**Figure 14f:**
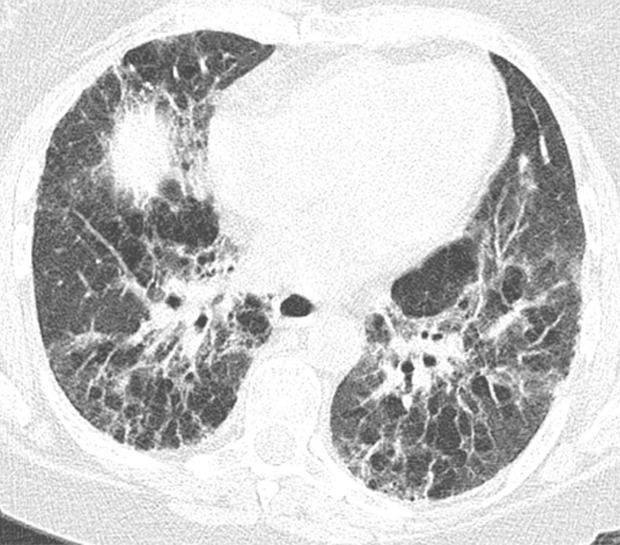
Fibrotic hypersensitivity pneumonitis. **(a–c)** Axial inspiratory CT scans demonstrate substantial ground-glass abnormality, mild centrilobular nodularity, and moderate mosaic attenuation, in addition to peribronchovascular and lower lung–predominant fibrosis. **(d–f)** Axial expiratory CT images confirm air trapping as the cause of mosaic attenuation. The degree of ground-glass abnormality, the degree of air trapping, and the peribronchovascular predominance of fibrosis would result in this case being categorized as most consistent with a nonidiopathic pulmonary fibrosis diagnosis.

### Sarcoidosis

All four stages of sarcoidosis tend to demonstrate an upper lung predominance at thin-section CT. Additionally, the fibrosis tends to demonstrate a more central or peribronchovascular distribution and radiate outward from the superiorly retracted hila with substantial upper lung volume loss, all features that should point toward a non-IPF diagnosis (Fig 15). One should be aware that small airways disease manifesting as air trapping at thin-section CT is commonly seen in sarcoidosis, not just HP (22).

**Figure 15a:**
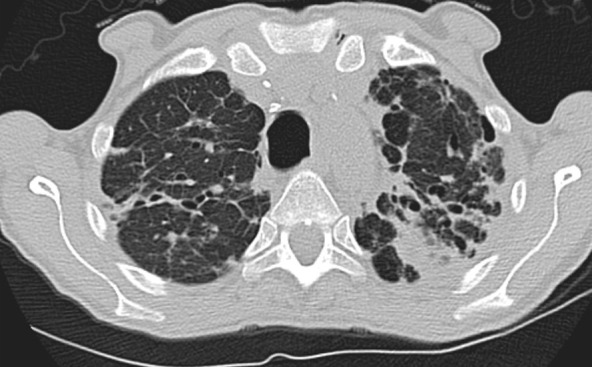
Advanced fibrotic sarcoidosis. **(a–c)** Axial inspiratory CT and **(d)** coronal inspiratory CT images demonstrate mid to upper lung–predominant pulmonary fibrosis with marked architectural distortion, associated lung nodularity, and some mosaic attenuation. Note also the calcified mediastinal lymph nodes. This case would be categorized as most consistent with a nonidiopathic pulmonary fibrosis diagnosis based on these features.

**Figure 15b:**
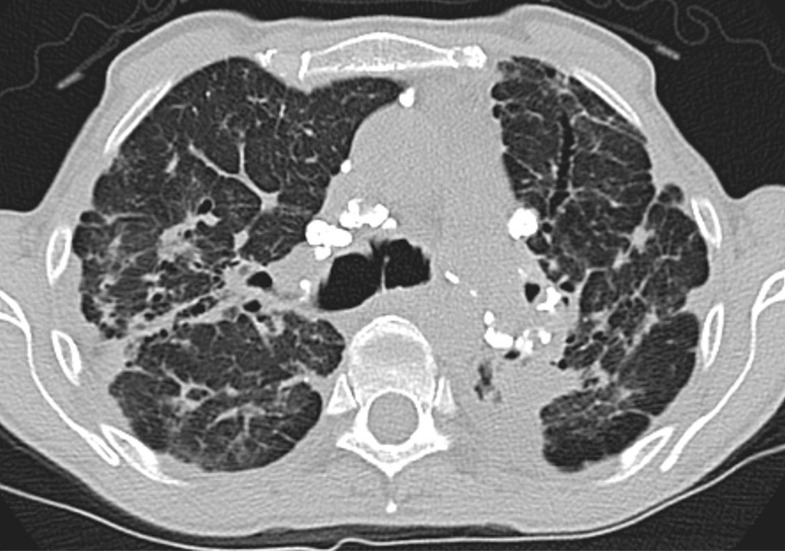
Advanced fibrotic sarcoidosis. **(a–c)** Axial inspiratory CT and **(d)** coronal inspiratory CT images demonstrate mid to upper lung–predominant pulmonary fibrosis with marked architectural distortion, associated lung nodularity, and some mosaic attenuation. Note also the calcified mediastinal lymph nodes. This case would be categorized as most consistent with a nonidiopathic pulmonary fibrosis diagnosis based on these features.

**Figure 15c:**
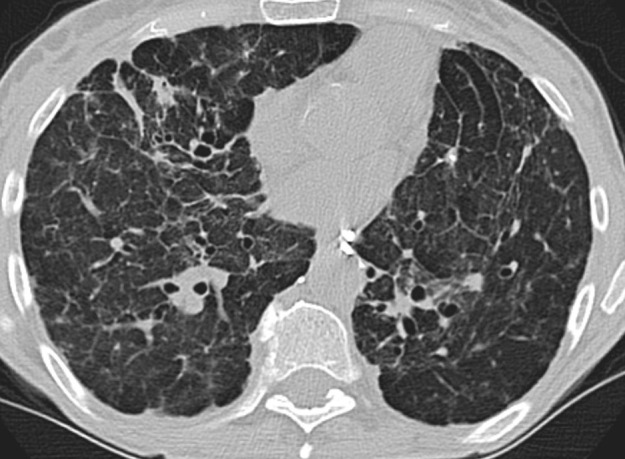
Advanced fibrotic sarcoidosis. **(a–c)** Axial inspiratory CT and **(d)** coronal inspiratory CT images demonstrate mid to upper lung–predominant pulmonary fibrosis with marked architectural distortion, associated lung nodularity, and some mosaic attenuation. Note also the calcified mediastinal lymph nodes. This case would be categorized as most consistent with a nonidiopathic pulmonary fibrosis diagnosis based on these features.

**Figure 15d:**
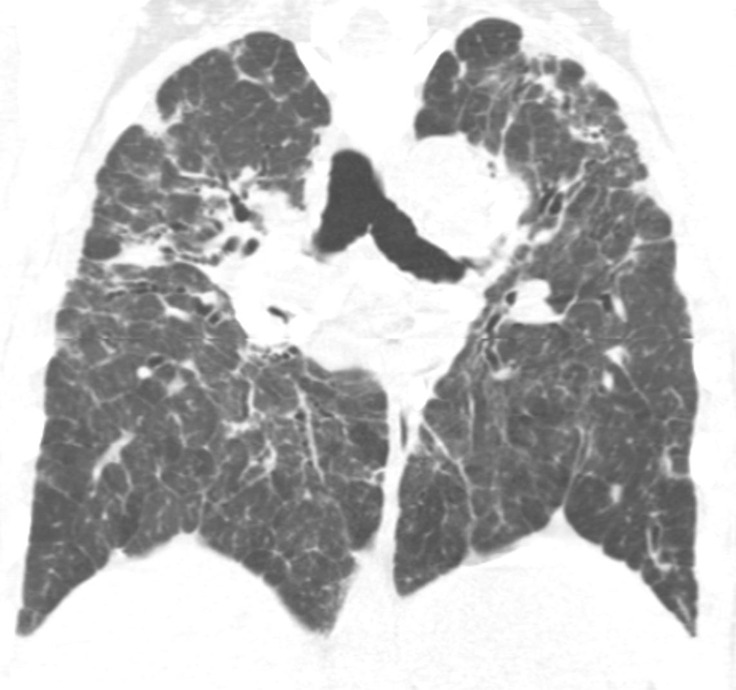
Advanced fibrotic sarcoidosis. **(a–c)** Axial inspiratory CT and **(d)** coronal inspiratory CT images demonstrate mid to upper lung–predominant pulmonary fibrosis with marked architectural distortion, associated lung nodularity, and some mosaic attenuation. Note also the calcified mediastinal lymph nodes. This case would be categorized as most consistent with a nonidiopathic pulmonary fibrosis diagnosis based on these features.

### Idiopathic Pleuroparenchymal Fibroelastosis

**Figure 16:**
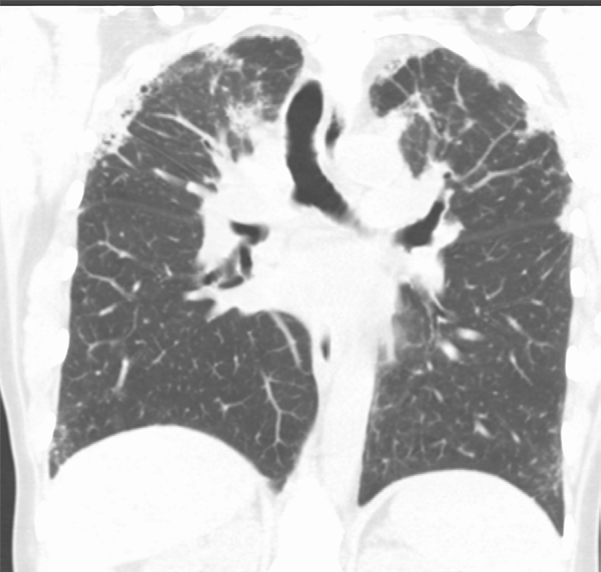
Pleuroparenchymal fibroelastosis in a 48-year-old woman. Coronal CT image shows dense subpleural consolidative abnormality with traction bronchiectasis and marked upper lobe volume loss.

Upper lung–predominant fibrosis with substantial associated apical pleural thickening characterizes the rare condition of idiopathic pleuroparenchymal fibroelastosis (Fig 16). There is a dense subpleural consolidation with associated traction bronchiectasis and upper lobe volume loss. The distribution in these cases should point away from IPF as a diagnosis (2). However, this entity can also be seen in conjunction with a concomitant UIP pattern.

### Acute Exacerbation of UIP

Accelerated deterioration or acute exacerbation of UIP and IPF typically manifests with a short prodrome of 1 to 2 months with progressive dyspnea or cough. Histopathologically, it represents diffuse alveolar damage and/or organizing pneumonia superimposed on UIP. As such, thin-section CT demonstrates bilateral ground-glass opacities and/or consolidations that mimic heart failure or opportunistic infection superimposed on the preexisting underlying fibrosis pattern (Fig 17). Understanding the clinical scenario of the patient undergoing imaging is critical to avoid miscategorizing the patient’s fibrosis pattern. For example, almost all patients undergoing an acute exacerbation of IPF would have their imaging pattern diagnosed as “CT features most consistent with a non-IPF diagnosis” on the basis of the existing guidelines due to the degree of ground-glass or consolidative abnormality present. However, if one understands the acute nature of the patient’s current illness, the possibility of acute exacerbation can be suggested, even if no prior imaging is available for comparison (23,24). Acute exacerbation may also occur with other fibrotic lung diseases, including chronic HP and NSIP.

**Figure 17a:**
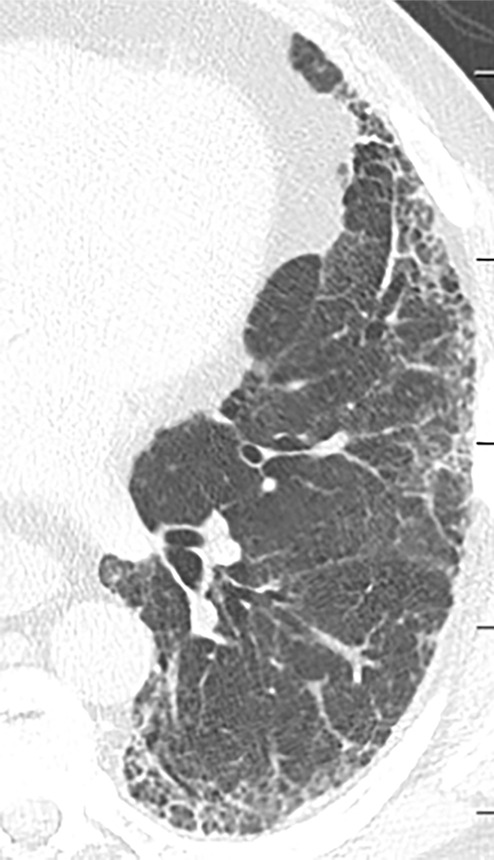
Acute exacerbation of idiopathic pulmonary fibrosis. **(a)** Zoomed view of axial CT demonstrates a probable usual interstitial pneumonia pattern in a patient with idiopathic pulmonary fibrosis. **(b)** Zoomed view of axial CT obtained after rapid deterioration in symptoms demonstrates CT findings of acute exacerbation with progression of reticular abnormality and marked new superimposed ground-glass opacity. Other causes of airspace disease including infection were clinically excluded. **(c)** Zoomed view of axial CT obtained after resolution of acute exacerbation confirms that the background fibrosis has substantially progressed.

**Figure 17b:**
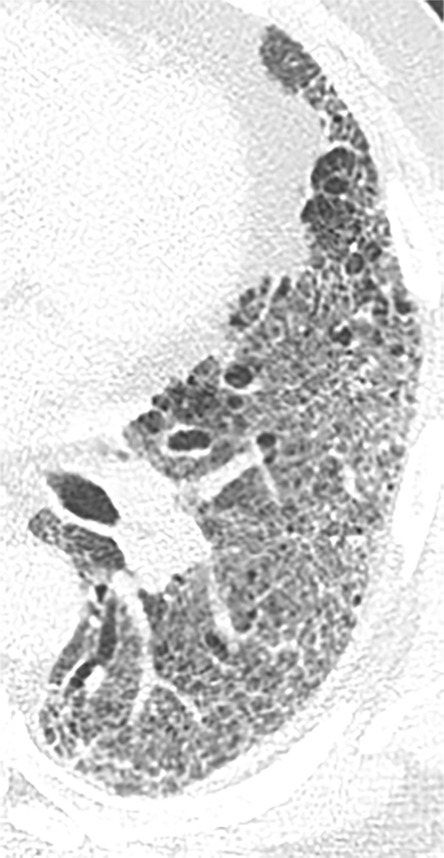
Acute exacerbation of idiopathic pulmonary fibrosis. **(a)** Zoomed view of axial CT demonstrates a probable usual interstitial pneumonia pattern in a patient with idiopathic pulmonary fibrosis. **(b)** Zoomed view of axial CT obtained after rapid deterioration in symptoms demonstrates CT findings of acute exacerbation with progression of reticular abnormality and marked new superimposed ground-glass opacity. Other causes of airspace disease including infection were clinically excluded. **(c)** Zoomed view of axial CT obtained after resolution of acute exacerbation confirms that the background fibrosis has substantially progressed.

**Figure 17c:**
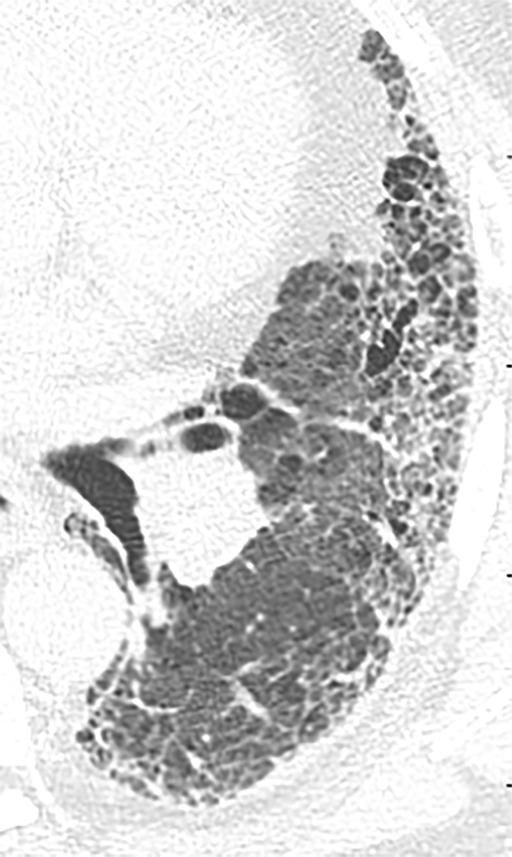
Acute exacerbation of idiopathic pulmonary fibrosis. **(a)** Zoomed view of axial CT demonstrates a probable usual interstitial pneumonia pattern in a patient with idiopathic pulmonary fibrosis. **(b)** Zoomed view of axial CT obtained after rapid deterioration in symptoms demonstrates CT findings of acute exacerbation with progression of reticular abnormality and marked new superimposed ground-glass opacity. Other causes of airspace disease including infection were clinically excluded. **(c)** Zoomed view of axial CT obtained after resolution of acute exacerbation confirms that the background fibrosis has substantially progressed.

### Interstitial Pneumonia with Autoimmune Features

Interstitial pneumonia is a common feature in patients with CTD, typically manifesting in conjunction with an established CTD diagnosis. However, ILD can occasionally manifest as the first or sole manifestation of a CTD. The ERS and ATS task force chose the term *interstitial pneumonia with autoimmune features* to describe patients who have idiopathic interstitial pneumonia with findings from defined clinical, serologic, and morphologic domains that indicate an underlying systemic autoimmune process but who do not meet diagnostic criteria of a specific CTD. Suggestive radiologic patterns include the following: NSIP, organizing pneumonia, and lymphoid interstitial pneumonia (25).

### Combined Pulmonary Fibrosis and Emphysema

**Figure 18:**
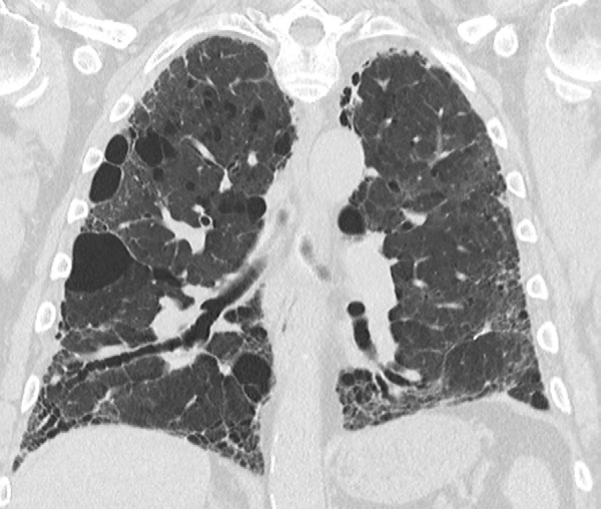
Combined pulmonary fibrosis and emphysema. Coronal CT image demonstrates upper lung–predominant centrilobular and paraseptal emphysema with additional lower lung, peripheral-predominant reticular abnormality, traction bronchiectasis, and honeycomb cyst formation.

Many patients with IPF are smokers or former smokers. Because of this smoking history, smoking-related changes are often present on pulmonary fibrosis imaging, including the presence of emphysema, enlargement of the airspaces with reticulation, respiratory bronchiolitis, smoking-related interstitial fibrosis, and desquamative interstitial pneumonia (3). The term *combined pulmonary fibrosis and emphysema* is used to describe patients with upper lobe–predominant emphysema and lower lobe–predominant pulmonary fibrosis (Fig 18). These patients tend to have a different clinical, physiologic, and radiologic outcome when compared with patients with either pulmonary fibrosis or emphysema alone, and therefore this entity should be recognized at imaging, as it can change patient treatment.

### Histologic Patterns in UIP, NSIP, and Fibrotic HP

Histologic evaluation has been very helpful for defining and distinguishing among the fibrosing interstitial pneumonias. Typical features of UIP include remodeling of lung parenchymal architecture by predominantly subpleural and paraseptal fibrosis (Fig 19). Fibroblastic foci are characteristic, and honeycombing may be seen. The term *temporal heterogeneity* is often used, referring to the fact that areas of dense fibrosis, early fibrosis, and normal lung may be seen in a single microscopic field (Fig 1). In contrast, NSIP is characterized by a spatially uniform pattern of expansion of alveolar septa by inflammatory and/or fibrotic cells, which is temporally homogeneous (Fig 20). In fibrotic HP, the fibrotic abnormality tends to predominate in the centrilobular region, and poorly formed granulomas or patches of organizing pneumonia may be present (Fig 21).

**Figure 19a:**
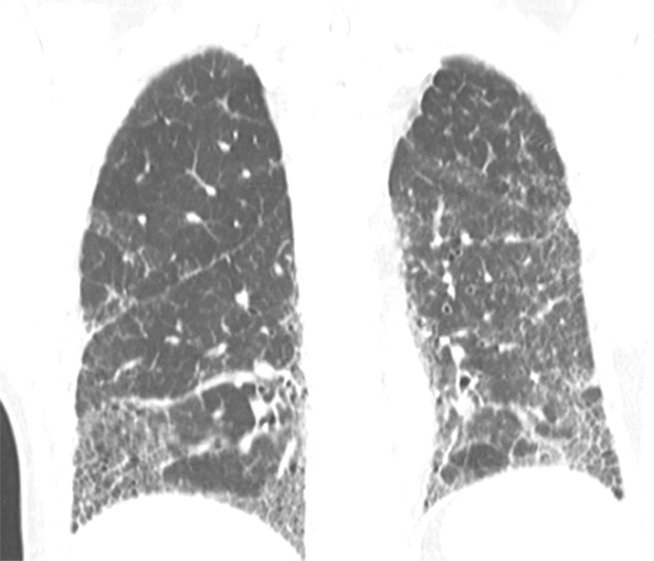
CT-histologic correlation in usual interstitial pneumonia. **(a)** Coronal CT image shows reticular abnormality and mild honeycombing with subpleural and lower lung predominance, typical of usual interstitial pneumonia. **(b)** Photomicrograph from histologic examination shows remodeling of the lung architecture by predominantly subpleural and paraseptal dense fibrosis, with scattered fibroblastic foci (blue arrow). Areas of normal lung are also seen, mainly in the centrilobular region, indicating temporal heterogeneity. (Histologic image courtesy of Rosane Duarte Achcar, MD, National Jewish Health.)

**Figure 19b:**
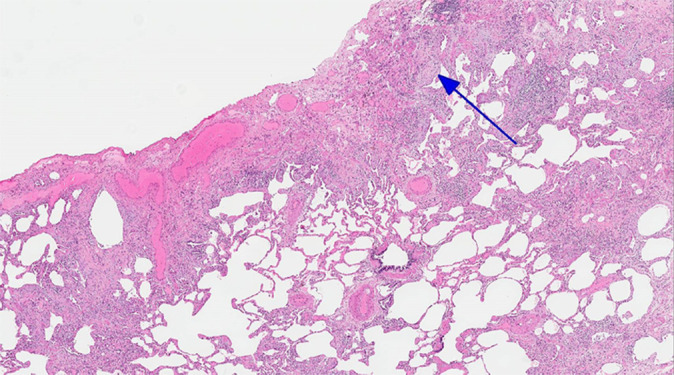
CT-histologic correlation in usual interstitial pneumonia. **(a)** Coronal CT image shows reticular abnormality and mild honeycombing with subpleural and lower lung predominance, typical of usual interstitial pneumonia. **(b)** Photomicrograph from histologic examination shows remodeling of the lung architecture by predominantly subpleural and paraseptal dense fibrosis, with scattered fibroblastic foci (blue arrow). Areas of normal lung are also seen, mainly in the centrilobular region, indicating temporal heterogeneity. (Histologic image courtesy of Rosane Duarte Achcar, MD, National Jewish Health.)

**Figure 20a:**
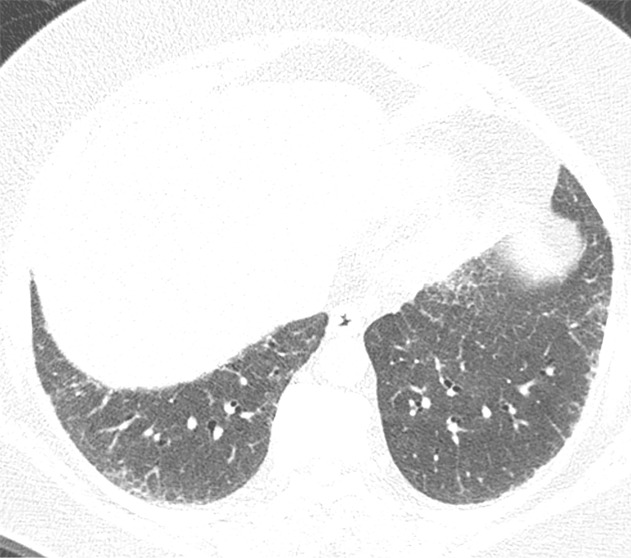
CT-histologic correlation in nonspecific interstitial pneumonia. **(a)** Axial CT image shows predominant ground-glass abnormality, with mild subpleural reticular abnormality. **(b)** Histologic image shows a diffuse homogeneous process with expansion of the alveolar septa by chronic inflammation and patchy interstitial scarring, unassociated with honeycomb change or substantial remodeling of the underlying lung architecture. In contrast to UIP, the abnormality is temporally homogeneous. (Hematoxylin-eosin stain) (Histologic image courtesy of Rosane Duarte Achcar, MD, National Jewish Health.)

**Figure 20b:**
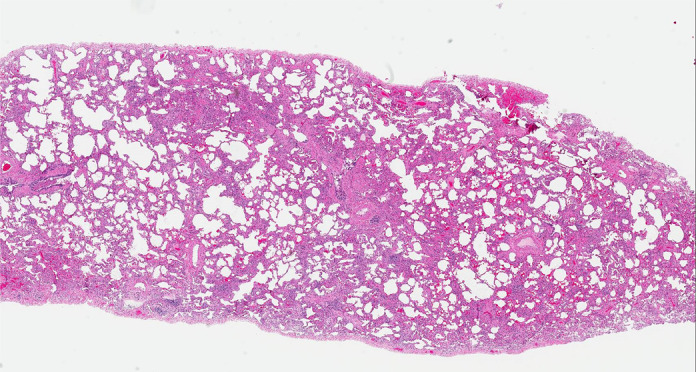
CT-histologic correlation in nonspecific interstitial pneumonia. **(a)** Axial CT image shows predominant ground-glass abnormality, with mild subpleural reticular abnormality. **(b)** Histologic image shows a diffuse homogeneous process with expansion of the alveolar septa by chronic inflammation and patchy interstitial scarring, unassociated with honeycomb change or substantial remodeling of the underlying lung architecture. In contrast to UIP, the abnormality is temporally homogeneous. (Hematoxylin-eosin stain) (Histologic image courtesy of Rosane Duarte Achcar, MD, National Jewish Health.)

**Figure 21a:**
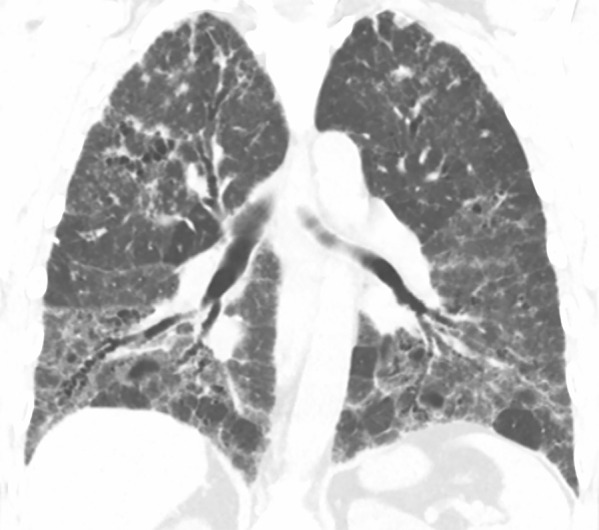
CT-histologic correlation in fibrotic hypersensitivity pneumonitis (HP). **(a)** Coronal CT image shows lower lung–predominant reticular and ground-glass abnormality. The findings of mosaic attenuation and peribronchovascular predominance are highly suggestive of fibrotic HP. **(b)** Photomicrograph from histologic examination shows centrilobular scarring compatible with fibrotic HP. (Hematoxylin-eosin stain) **(c)** Peribronchiolar poorly formed nonnecrotizing granulomas are also present, highly suggestive of HP. (Hematoxylin-eosin stain) (Histologic images courtesy of Rosane Duarte Achcar, MD, National Jewish Health.)

**Figure 21b:**
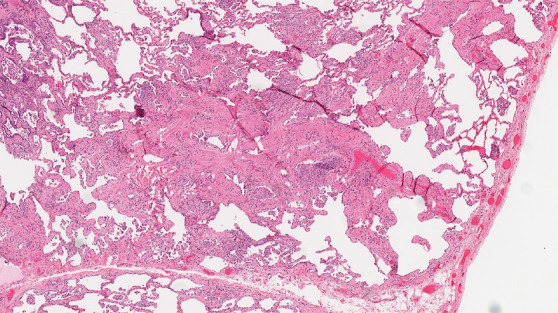
CT-histologic correlation in fibrotic hypersensitivity pneumonitis (HP). **(a)** Coronal CT image shows lower lung–predominant reticular and ground-glass abnormality. The findings of mosaic attenuation and peribronchovascular predominance are highly suggestive of fibrotic HP. **(b)** Photomicrograph from histologic examination shows centrilobular scarring compatible with fibrotic HP. (Hematoxylin-eosin stain) **(c)** Peribronchiolar poorly formed nonnecrotizing granulomas are also present, highly suggestive of HP. (Hematoxylin-eosin stain) (Histologic images courtesy of Rosane Duarte Achcar, MD, National Jewish Health.)

**Figure 21c:**
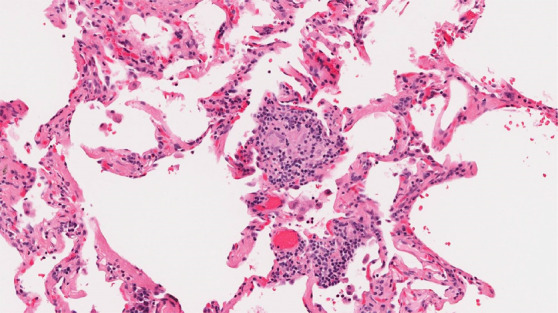
CT-histologic correlation in fibrotic hypersensitivity pneumonitis (HP). **(a)** Coronal CT image shows lower lung–predominant reticular and ground-glass abnormality. The findings of mosaic attenuation and peribronchovascular predominance are highly suggestive of fibrotic HP. **(b)** Photomicrograph from histologic examination shows centrilobular scarring compatible with fibrotic HP. (Hematoxylin-eosin stain) **(c)** Peribronchiolar poorly formed nonnecrotizing granulomas are also present, highly suggestive of HP. (Hematoxylin-eosin stain) (Histologic images courtesy of Rosane Duarte Achcar, MD, National Jewish Health.)

## Final Diagnosis of IPF for a Given Thin-Section CT Pattern

Taking these thin-section CT patterns into consideration, there are several methods for arriving at a diagnosis of IPF (3):

CT pattern of typical UIP or probable UIP.—If clinical scenario is consistent with IPF, biopsy is unnecessary. If clinical scenario is not typical for IPF (eg, patient aged < 60, relevant inhalational exposures), biopsy is necessary.

CT pattern indeterminate for UIP or CT features most consistent with an alternative diagnosis.—Biopsy should be considered regardless of clinical scenario.

Biopsy is unavailable.—A working diagnosis of IPF can be established if *(a)* there is a progressive fibrosing interstitial pneumonia, and *(b)* there is no viable alternative diagnosis. Any such working diagnosis of IPF should be reviewed regularly.

When considering biopsy, it is important to remember that open lung biopsy is not without patient risk. Although some patients may need biopsy to confirm a confidence diagnosis, biopsy may not be appropriate on the basis of their risk profile. Also, biopsy may not be of benefit unless there are identifiable treatment changes that would be affected by confirming a diagnosis.

## Reporting Thin-Section CT Scans for Diffuse Lung Disease

Taking the above information into consideration, each report for evaluation of lung fibrosis should include the presence or absence of the features defined in the previous text, with the ultimate goal of identifying whether a UIP pattern is present and the interpreter’s confidence level (UIP, probable UIP, etc). It is important to comment on the presence of new ground-glass opacities as well as to evaluate for disease progression, not only comparing with the most recent prior examination, but also with older studies (if available). Other commonly observed findings associated with IPF and other diffuse lung diseases should also be reported as they may frequently guide toward an alternative diagnosis such as the following:

1. Emphysema, which can be a confounding factor in patients with a UIP pattern at CT. The extent and relative severity should be reported, as it influences patient treatment and prognosis (26).
2. Pleural effusion, esophageal dilatation, or pericardial abnormality, which regardless of confidence of UIP pattern should raise the possibility of underlying CTD.
3. Hiatal hernia along with esophageal thickening or dilatation, which are important findings, as chronic aspiration is an underreported but common cause of fibrosis involving the basilar lungs and can also contribute to worsening fibrosis.
4. Cardiac chamber and pulmonary arterial size, as the presence of pulmonary hypertension can be an important prognostic factor.
5. Lymphadenopathy and pulmonary nodules, which are common findings with underlying ILD; however, a new or enlarging pulmonary nodule or enlarging lymph nodes should be critically considered, as the risk of developing malignancy (particularly lung cancer and lymphoma) is elevated in this patient population.

Please see Tables 4 and 5, which provide sample templates for potential use. Table 4 highlights a highly structured comprehensive report for evaluation of potential IPF cases. Use and implementation of such a highly structured report ensures that all key terminology is used and allows for data mining of these reports for further research efforts. Table 5 provides a sample report that is more focused and could be more easily integrated into existing templates at most institutions.

**Table 4:**
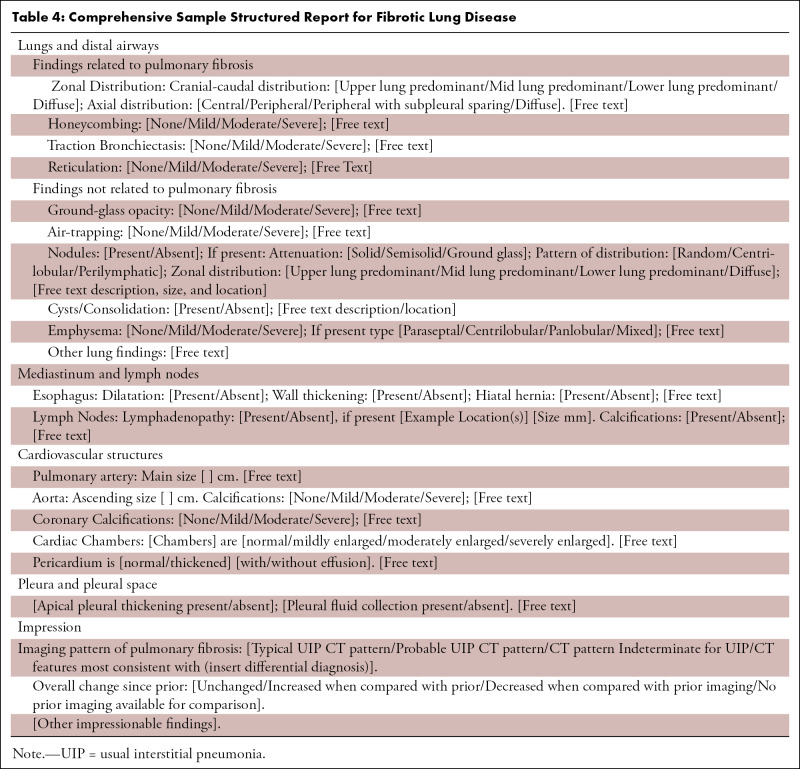
Comprehensive Sample Structured Report for Fibrotic Lung Disease

**Table 5:**
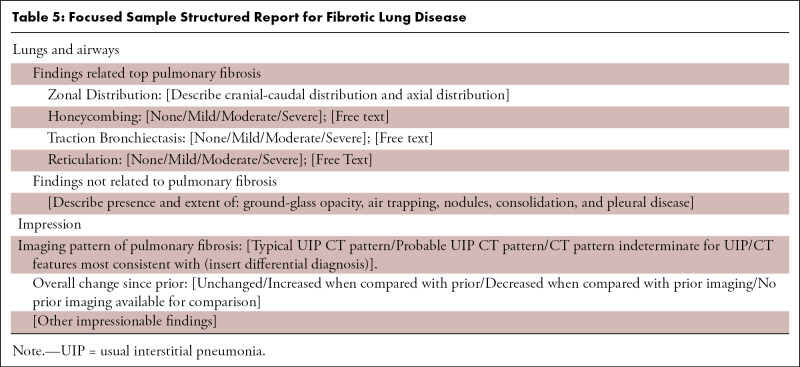
Focused Sample Structured Report for Fibrotic Lung Disease

## Radiologic and Pathologic Communication and the Role of Multidisciplinary Discussion

In patients suspected of having pulmonary fibrosis, the initial steps in clinical work are to assess for any potential known causes of pulmonary fibrosis. As such, integration of this clinical information into the diagnostic process is key and when combined with the thin-section CT pattern present, frequently alleviates the need for invasive lung biopsies. Both the Fleischner Society guidelines and the ATS/ERS/JRS/ALAT IPF guidelines provide algorithms for approaching a diagnosis (3,6). Thin-section CT is necessary for key decision-making points and should be performed in all cases where IPF is suspected. In addition to thin-section CT, other testing, including pulmonary function testing, bronchoscopy, and serologic evaluation, plays a key role in evaluation and may provide clues to a specific diagnosis. Although some guidelines exist for diagnosis of CTDs, these diagnoses frequently benefit from expert consultation with rheumatologists who can provide key insight into disease behavior and serologic evaluation of these patients (27). Additionally, multiple pathologies can exist in a single patient and are most frequently seen in the setting of underlying CTD such as NSIP and organizing pneumonia in patients with myositis. Finally, there is substantial interobserver variability among pathologists in assessment of diffuse lung disease, which can be mitigated at least partially by providing adequate clinical and radiologic information to the interpreting pathologist (28).

It is recommended that multidisciplinary diagnosis groups should consist of clinicians, radiologists, and pathologists with experience in ILD and be conducted in a face-to-face public forum format, with a teleconference format a reasonable alternative if an in-person conference format is impractical. These groups have been shown to improve patient outcomes on a case-by-case basis by reviewing hard-to-categorize clinical, radiologic, and histologic findings, especially important when there is discordant clinical, pathologic, and/or radiologic findings (3,6). It has been reported that use of multidisciplinary discussion can lead to a change in diagnosis in up to 30% of cases (many of which are cases demonstrating discordance between imaging and pathology), therefore preventing delay in diagnosis or initiation of therapy, preventing incorrect therapy application, and preventing unneeded or inappropriate additional testing (6).

## Conclusions

Thin-section CT is the primary diagnostic imaging tool for initial categorization and follow-up imaging of IPF. As such, radiologists evaluating these cases should be familiar with the diagnostic criteria of the UIP pattern as well as the findings seen in other causes of pulmonary fibrosis. Understanding these imaging patterns greatly facilitates multidisciplinary discussions that serve as the reference standard for diagnosis and management of IPF.
